# A versatile synthetic strategy for macromolecular cages: intramolecular consecutive cyclization of star-shaped polymers†
†Electronic supplementary information (ESI) available: Experimental procedures and additional data (^1^H NMR, MALDI-TOF MS, SEC, DSC, SAXS, and WAXD). See DOI: 10.1039/c8sc04006k


**DOI:** 10.1039/c8sc04006k

**Published:** 2018-10-11

**Authors:** Yoshinobu Mato, Kohei Honda, Kenji Tajima, Takuya Yamamoto, Takuya Isono, Toshifumi Satoh

**Affiliations:** a Graduate School of Chemical Sciences and Engineering , Hokkaido University , Sapporo 060-8628 , Japan; b Division of Applied Chemistry , Faculty of Engineering , Hokkaido University , Sapporo 060-8628 , Japan . Email: isono.t@eng.hokudai.ac.jp ; Email: satoh@eng.hokudai.ac.jp

## Abstract

Cage-shaped polymers, or “macromolecular cages”, are of great interest as the macromolecular analogues of molecular cages because of their various potential applications in supramolecular chemistry and materials science. However, the systematic synthesis of macromolecular cages remains a great challenge. Herein, we describe a robust and versatile synthetic strategy for macromolecular cages with defined arm numbers and sizes based on the intramolecular consecutive cyclization of highly reactive norbornene groups attached to each end of the arms of a star-shaped polymer precursor. The cyclizations of three-, four-, six-, and eight-armed star-shaped poly(ε-caprolactone)s (PCLs) bearing a norbornenyl group at each arm terminus were effected with Grubbs' third generation catalyst at high dilution. ^1^H NMR, SEC, and MALDI-TOF MS analyses revealed that the reaction proceeded to produce the desired macromolecular cages with sufficient purity. The molecular sizes of the macromolecular cages were controlled by simply changing the molecular weight of the star-shaped polymer precursors. Systematic investigation of the structure–property relationships confirmed that the macromolecular cages adopt a much more compact conformation, in both the solution and bulk states, as compared to their linear and star-shaped counterparts. This synthetic approach marks a significant advance in the synthesis of complex macromolecular architectures and provides a platform for novel applications using cage-shaped molecules with polymer frameworks.

## Abstract 2

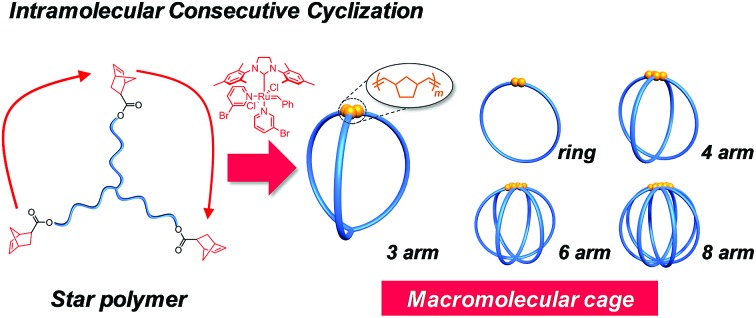
Intramolecular consecutive cyclization was established as a versatile and robust strategy to synthesize macromolecular cages.

## Introduction

Molecular cages have drawn attention as intriguing chemical research subjects because of their structural uniqueness, featuring three-dimensional cavities inside their molecular frameworks.^1^–^4^ Owing to such distinctive architectures, cage molecules have been utilized for a diverse range of applications, such as nanocapsules for relatively large molecules,^5^,^6^ templates for the size-controlled synthesis of metal nanoparticles,^7^ and nanoflasks for catalytic reactions.^8^,^9^ Cage molecules can be constructed by either the formation of covalent bonds or self-assembly through non-covalent bonding interactions. A number of small organic cages such as cryptands have been synthesized mainly by the former approach.^10^,^11^ On the other hand, the latter approach has recently proven to be a powerful means to construct giant cages such as DNA and protein cages as well as 3D metallocages with a molecular size range of 1–50 nm.^12^–^15^ Remarkably, the robustness of the self-assembly approach has enabled the creation of novel cage molecules possessing various sizes, topologies, and functionalities.^1^,^16^ However, the non-covalent bonding interactions are highly sensitive to external conditions, so that the self-assembled cages are relatively fragile upon exposure to chemical/physical stimuli including pH, temperature, and solvent polarity. To further extend the possible applications of cage molecules, a novel covalent-bond-forming strategy for the systematic synthesis of cage molecules with tunable functionality and controllable cavity size is highly desired.

**Fig. 1:**
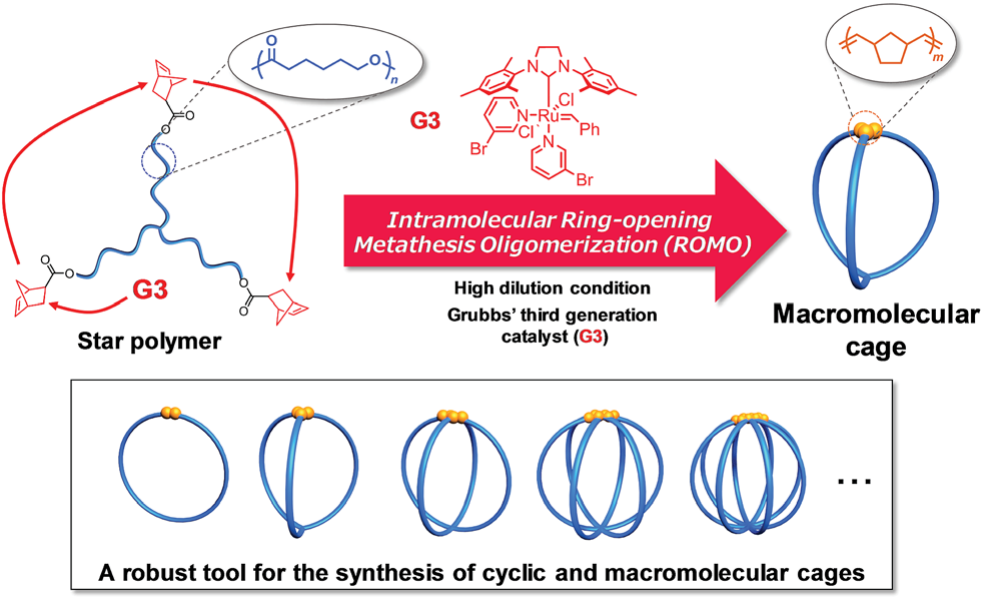
Schematic illustration of the synthetic strategy for macromolecular cages through intramolecular consecutive ROMO mediated by G3.

To achieve this goal, we envisioned the use of synthetic polymers as building blocks for the cage-shaped framework. A major advantage of a synthetic polymer is that a cage molecule of a targeted size can be readily synthesized by simply tuning its degree of polymerization. Moreover, we anticipated that the choice of comonomers and their sequence would endow the cage molecules with vast functional utility such as molecular recognition ability, external stimuli responsiveness, and the ability to self-assemble into higher-order structures.^17^–^20^ However, only limited efforts have been made thus far to prepare macromolecular cages, and therefore, a general synthetic strategy remains lacking. To date, macromolecular cages composed of up to four arms have been reported by the groups of Tezuka and Paik, and more recently, our own.^21^–^24^ Although each synthesis produced well-defined macromolecular cages, the laborious and multistep natures of these conventional syntheses present a practical limitation to the systematic synthesis of macromolecular cages with arm numbers greater than five. Consequently, the structures and properties of macromolecular cages, especially with respect to the molecular weight and arm number, have never been systematically studied. To achieve the systematic synthesis of macromolecular cages with varying arm numbers, we devised a novel synthetic approach which involves intramolecular cyclization in a chain-reaction manner to construct a multi-ring system. Such chain reaction-type cyclization would be ideal for the step-efficient synthesis of macromolecular cages with various arm numbers. As shown in Fig. 1, the intramolecular consecutive cyclization of star-shaped polymers bearing a polymerizable group at each chain end would enable the systematic synthesis of macromolecular cages.

To selectively produce the desired macromolecular cage, it is essential to suppress possible intermolecular propagation reactions by applying highly dilute conditions. Considering the extraordinarily high reactivity of ring-opening metathesis polymerization (ROMP) coupled with the Grubbs 3^rd^ generation catalyst (G3),^25^–^27^ the *exo*-norbornene group was selected as the polymerizable end group. Meanwhile, we employed poly(*ε*-caprolactone) (PCL) as the macromolecular framework of the cage due to its ease of preparation with sufficient chain-end fidelity and narrow dispersity (*Đ*, < 1.1).^28^ We herein demonstrate, for the first time, the systematic synthesis of macromolecular cages with varied arm numbers and molecular weights by the intramolecular ring-opening metathesis oligomerization (ROMO) of *exo*-norbornene groups attached to each chain end of the star-shaped PCLs. In addition, we demonstrate a comprehensive study of the correlation between the cage-shaped structures and polymer properties by employing a series of cage-shaped PCLs.

## Results and discussion

**Scheme 1:**
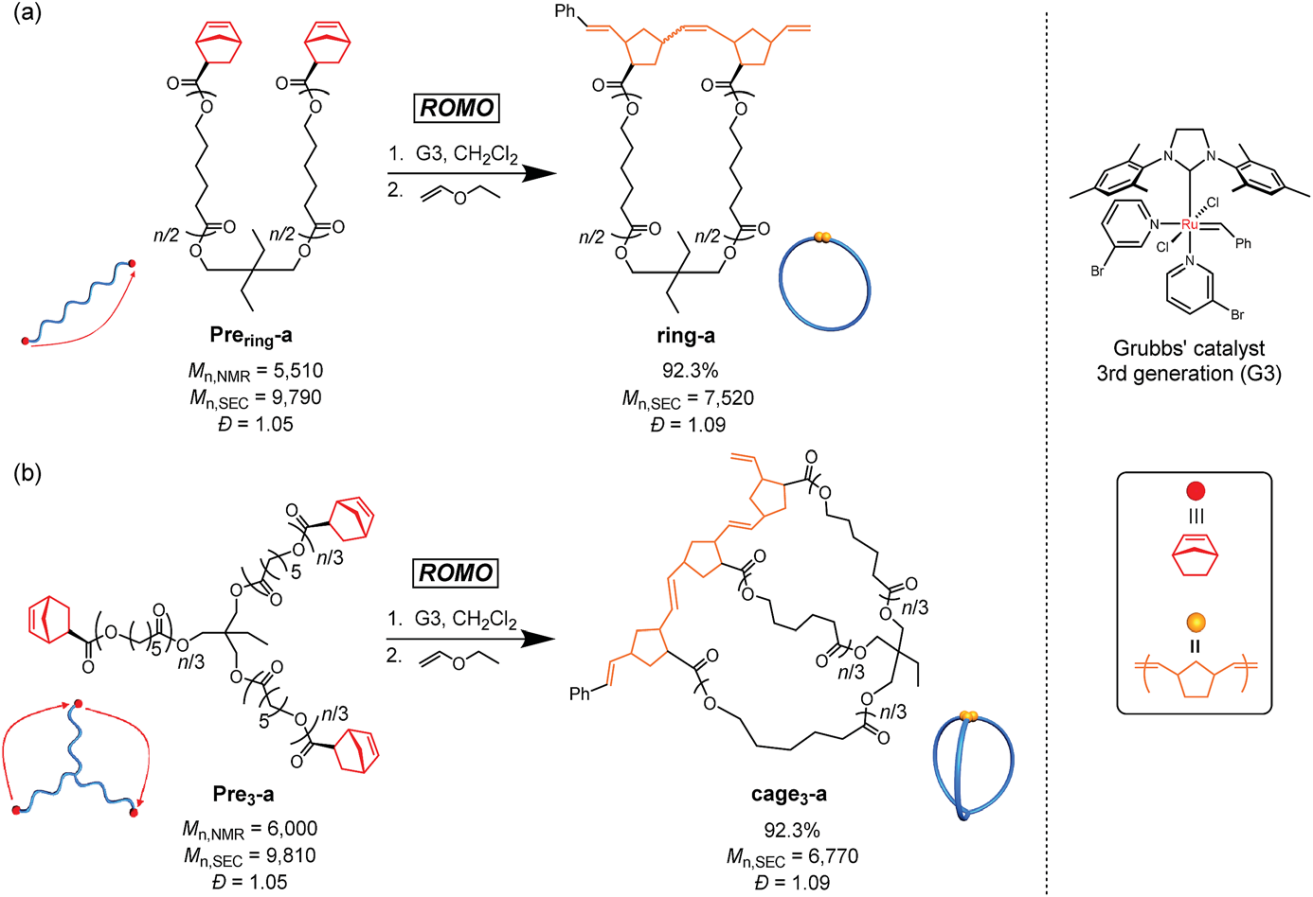
Synthesis of **ring-a** (a) and three-armed **cage_3_-a** (b) through intramolecular ring-opening metathesis oligomerization (ROMO) of the corresponding PCLs with a reactive norbornenyl group at each chain end.

As a model reaction system, we initially optimized the reaction conditions for the intramolecular consecutive ROMO of an *α*,*ω*-dinorbornenyl end-functionalized linear PCL precursor, **Pre_ring_-a**, to afford the corresponding monocyclic polymer **ring-a** (Scheme 1a). The G3-mediated ROMO of **Pre_ring_-a** (number-average molecular weight (*M*_n_) estimated by ^1^H NMR (*M*_n,NMR_) = 5510, *M*_n_ estimated by SEC-RI using PSt standards (*M*_n,SEC_) = 9790, *Đ* = 1.05; see Section S1-3 and Table S1 in the ESI† for more details) was conducted at high dilution (final polymer precursor concentration = 0.02 mM in CH_2_Cl_2_) to preferentially promote the desired intramolecular reaction rather than the undesired intermolecular one. To further minimize intermolecular coupling, the solution of **Pre_ring_-a** was added dropwise to the stirred G3 solution in CH_2_Cl_2_ with varying [**Pre_ring_-a**]_0_/[G3]_0_ ratios of 1/1, 1/2, 1/4, and 1/6. Notably, each reaction produced a soluble product, and ^1^H NMR analysis suggested quantitative consumption of the norbornenyl group (Fig. S1†). The molecular weight distribution of the product varies significantly depending upon the [**Pre_ring_-a**]_0_/[G3]_0_ ratio, as shown in Fig. S2.† The size exclusion chromatography (SEC) traces of the products obtained at the [**Pre_ring_-a**]_0_/[G3]_0_ ratios of 1/1, 1/2, and 1/4 exhibit two elution peak maxima in both higher and lower molecular weight regions with respect to that of **Pre_ring_-a**, indicating the formation of intramolecular propagation and intermolecular cyclization products, respectively.^29^ On the other hand, the product obtained at the [**Pre_ring_-a**]_0_/[G3]_0_ ratio of 1/6 exhibits a narrowly dispersed unimodal peak in a lower molecular weight region that would be assignable to the intramolecularly constructed product **ring-a**, as reported previously.^30^ The decrease in the apparent molecular weight after the ROMO with hydrodynamic volume change (0.75; calculated from the equation *M*_p,SEC(Pre_ring_)_/*M*_p,SEC(ring)_, where *M*_p,SEC_ is the peak-top molecular weight; Fig. S3†) is consistent with the literature range of 0.71–0.83 for other monocyclic polymers possessing comparable molecular weight.^31^–^33^ Thus, successful cyclic structure formation is demonstrated for the synthesis of **ring-a** with suppressed intermolecular propagation. Moreover, the ^1^H NMR and matrix-assisted laser desorption ionization time-of-flight (MALDI-TOF) mass spectra could be reasonably assigned to the expected chemical structure of **ring-a** (Fig. S4†). Hence, we found that a [precursor]_0_/[G3]_0_ ratio of 1/6 in combination with a slow addition technique can selectively promote intramolecular ROMO.

**Fig. 2:**
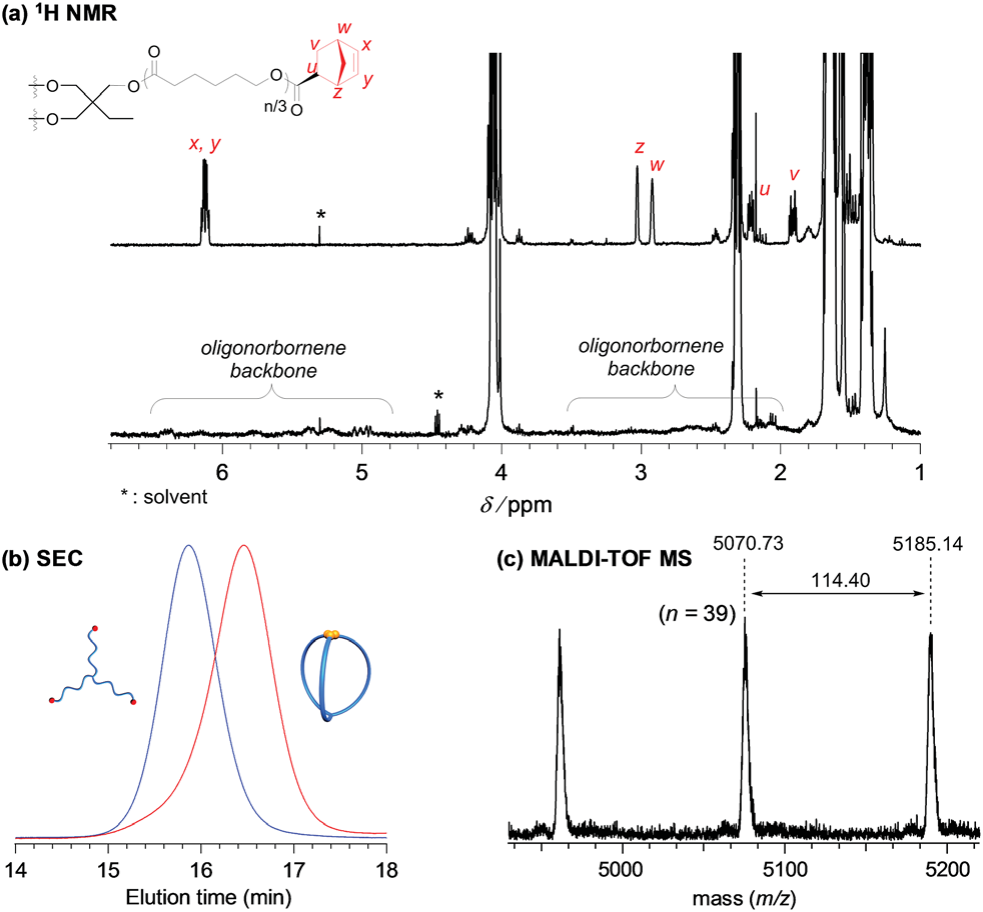
Structural analysis of the three-armed macromolecular cage (**cage_3_-a**). (a) ^1^H NMR spectra of **Pre_3_-a** (upper) and **cage_3_-a** (lower). (b) SEC traces of **Pre_3_-a** (*M*_n,NMR_ = 6000, *M*_n,SEC_ = 9810, *Đ* = 1.05; blue line) and **cage_3_-a** (before purification by preparative SEC; *M*_n,SEC_ = 6770, *Đ* = 1.09; red line) synthesized by intramolecular ROMO. (c) Expanded MALDI-TOF MS spectrum of **cage_3_-a** ranging from 4500 to 5200 Da.

Next, we synthesized the three-armed star-shaped PCL bearing a norbornenyl group at each end, **Pre_3_-a** (*M*_n,NMR_ = 6000, *M*_n,SEC_ = 9810, *Đ* = 1.05), which is subject to the envisioned intramolecular ROMO to give the corresponding three-armed macromolecular cage, **cage_3_-a** (Scheme 1b). The preparation of **Pre_3_-a** is successfully achieved in two steps: (i) diphenyl phosphate-catalyzed ring-opening polymerization of *ε*-caprolactone (*ε*-CL) using a commercially available triol initiator with the [*ε*-CL]_0_/[initiator]_0_ ratio of 50/1 and (ii) subsequent condensation reaction with excess (±)-*exo*-5-norbornene carboxylic acid (see Section S1-3†). The ^1^H NMR spectrum of the product clearly shows the signals attributed to the norbornenyl groups, indicating quantitative introduction of norbornenyl groups to each polymer end (Fig. 2a and S5†). The SEC traces of the obtained product retained monomodal features even after the condensation reaction (Fig. S6†).

With the optimized reaction conditions ([precursor]_0_/[G3]_0_ = 1/6; [precursor]_0_ = 0.02 mM) in hand, the intramolecular consecutive ROMO of **Pre_3_-a** was carried out to give **cage_3_-a** (Scheme 1b). In the ^1^H NMR spectrum of the product, no signals due to the norbornenyl group are detected, whereas signals attributable to the oligonorbornene backbone are observed near 1.05–3.25 and 4.95–6.60 ppm (Fig. 2a). Although the ^1^H NMR analysis revealed the completion of the reaction, several possible side reactions such as intermolecular polymerization and the multiple addition of G3 were also considered (Fig. S8†). To exclude the possibilities of such side reactions, SEC and MALDI TOF-MS analyses were performed to further verify the detailed structure of the product. The SEC trace of the obtained product is significantly shifted to the lower molecular region (*M*_n,SEC_ = 6770, *Đ* = 1.09) as compared to **Pre_3_-a** (*M*_n,SEC_ = 9810), implying the formation of the desired cage-shaped product with a smaller hydrodynamic volume (Fig. 2b and S6†).

**Fig. 3:**
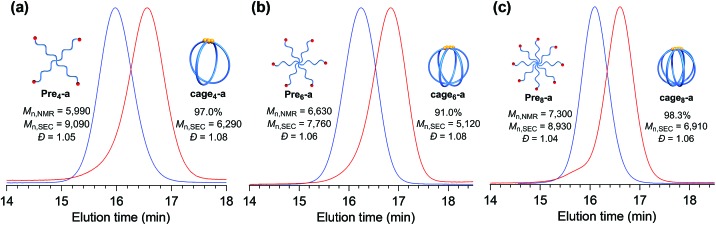
SEC traces for (a) four-, (b) six-, and (c) eight-armed star-shaped precursors (blue; **Pre_4_-a**, **Pre_6_-a**, and **Pre_8_-a**, respectively) and the macromolecular cages constructed *via* intramolecular ROMO (red; **cage_4_-a**, **cage_6_-a**, and **cage_8_-a**, respectively).

Furthermore, the monomodal elution peak is retained after the reaction, suggesting that the intermolecular reaction is highly suppressed. Although a small higher molecular weight shoulder, perhaps due to the dimer and trimer formed *via* the intermolecular propagation, was observed in the SEC trace, the purity of the desired product was calculated to be higher than 90% based on the elution peak area (Table S2†). Moreover, the MALDI-TOF mass spectrum shows only one set of peaks with a regular interval of 114.40 Da corresponding to the *ε*-CL monomer unit, which is a good indication that multiple G3 addition did not occur (Fig. 2c and S7†). Specifically, peaks due to possible multiple G3 adducts, such as the tadpole- (for example [M + Na]^+^ = 5059.97 Da, *n* = 38) and star-shaped polymers (for example [M + Na]^+^ = 5165.03 Da, *n* = 38, Fig. S8†), are not detected. In addition, an observed peak at *m*/*z* 5070.73 Da agrees with the calculated mass for the desired **cage_3_-a** with a degree of polymerization of 39 ([M + Na]^+^ = 5070.05 Da, *n* = 39). Overall, these data strongly confirm that the intramolecular consecutive ROMO using G3 produces **cage_3_-a** with sufficient purity. The optimized ROMO reaction conditions were also applicable to four-, six-, and eight-armed star-shaped PCLs bearing a norbornene at each chain end (**Pre_4_-a**, **Pre_6_-a**, and **Pre_8_-a**), which afforded the corresponding cage-shaped PCLs with varied arm numbers (**cage_4_-a**, **cage_6_-a**, and **cage_8_-a**, respectively) in good yields, typically in the range of 80–97% (Fig. 3, Tables S3–S5†). Each product was fully characterized by SEC, ^1^H NMR, and MALDI-TOF MS, which confirmed the successful synthesis of the macromolecular cages (Fig. S9–S18†). It is worth noting that narrowly dispersed macromolecular cages (*Đ* = 1.06–1.09) were obtained without obvious side reactions, despite the increase in the arm-numbers of the precursors. Although some of the obtained macromolecular cages showed high molecular shoulders in their SEC traces, the purity was calculated to be more than 89%, according to the SEC elution peak area. These results suggest that G3-mediated intramolecular ROMO proceeds in preference to the addition of a second G3 to other norbornenyl groups in the same molecule, preventing the formation of possible by-products such as the tadpole-shaped product. In addition, we have succeeded in controlling the molecular weight of a series of macromolecular cages in an *M*_n,NMR_ range of ∼6000–12 000 (Table 1) by simply employing star-shaped PCLs with different molecular weights. Hence, we have established a versatile yet robust synthetic strategy for macromolecular cages based on the intramolecular ROMO that enables the production of a series of macromolecular cages with controlled molecular weights and arm numbers.

**Table 1:** Molecular characteristics of cyclic polymers and macromolecular cages obtained by intramolecular ROMO

Macromolecular cage	*M*_n,SEC_ ^*a*^	*M*_w,MALS_ ^*b*^	*Đ* ^*a*^	*D*_h_ ^*c*^ (nm)	Yield (%)
**ring-a**	7520	6280	1.09	4.6	92.3
**ring-b**	11 700	8890	1.09	5.8	92.3
**ring-c**	15 200	11 600	1.09	6.8	91.6
**cage_3_-a**	6770	7550	1.09	4.4	92.3
**cage_3_-b**	9360	9370	1.09	5.0	80.0
**cage_3_-c**	11 500	10 200	1.09	5.6	84.0
**cage_4_-a**	6290	7420	1.08	4.2	97.0
**cage_4_-b**	9590	9840	1.08	5.0	94.0
**cage_4_-c**	10 700	11 800	1.08	5.6	91.0
**cage_6_-a**	5120	7950	1.08	3.8	91.0
**cage_6_-b**	8570	10 900	1.09	5.0	98.7
**cage_6_-c**	10 700	12 800	1.07	5.2	91.2
**cage_8_-a**	6910	8180	1.06	3.8	98.3
**cage_8_-b**	9130	10 300	1.08	4.6	97.7
**cage_8_-c**	12 000	14 300	1.06	5.6	84.7

^*a*^Determined by SEC in THF using PSt standards.

^*b*^Weight-average absolute molecular weight (*M*_w,MALS_) was estimated by SEC-MALS-Visco in THF.

^*c*^Weight-average hydrodynamic diameter (*D*_h_) was determined through SEC-MALS-Visco measurements in THF by the following equations: *D*_h_ = 2*R*_h_ = 2(3*V*_h_/4π)^1/3^ where *V*_h_ (hydrodynamic volume) was calculated using the Einstein–Simha equation (*V*_h_ = *M*_w,MALS_[*η*]/2.5*N*_A_, where *N*_A_ is Avogadro's number).

**Fig. 4:**
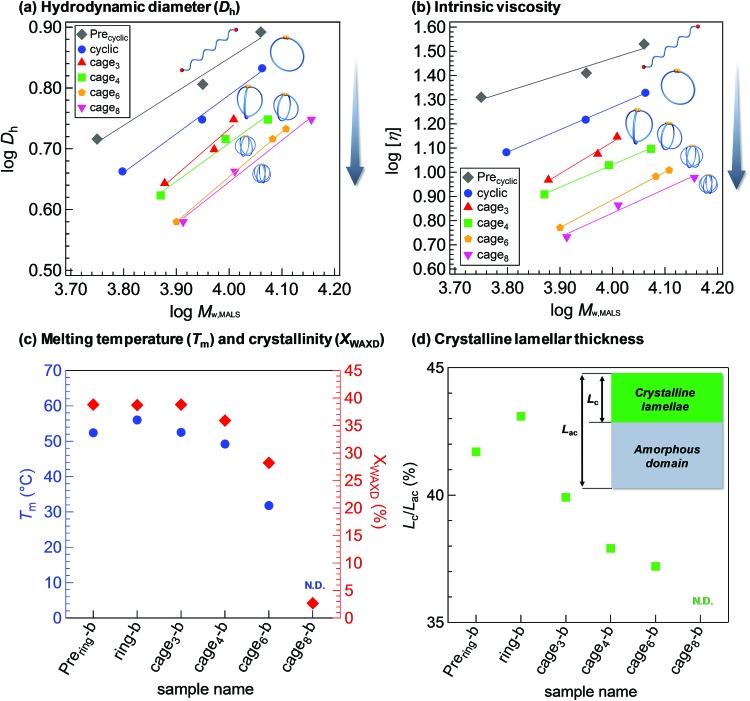
Structure–property relationships in macromolecular cage systems. (Panels a and b) Double logarithmic plots of *M*_w,MALS_*versus D*_h_ and [*η*] for linear (**Pre_ring_**) and monocyclic (**ring**) (eluent, THF) polymers. (Panels c and d) Plots of *T*_m_ (blue), *X*_WAXD_ (red), and (d) *L*_c_/*L*_ac_ (green) *versus* arm numbers of linear, monocyclic polymers, and macromolecular cages (all the samples had a *M*_n,NMR_ of *ca.* 9000; from left to right, **Pre_ring_-b**, **ring-b**, **cage_3_-b**, **cage_4_-b**, **cage_6_-b**, and **cage_8_-b**). The inset in panel (d) illustrates the model structure of the long period of the PCL crystal which consists of a crystalline lamellar and an amorphous domain with thicknesses of *L*_c_ and *L*_ac_, respectively. The *T*_m_ and *L*_c_/*L*_ac_ for **cage_8_-b** were not determined (N.D.) due to its poor crystallinity.

Owing to the lack of a universal synthetic strategy, the polymer properties associated with a cage-shaped architecture have never been systematically evaluated, although a comprehensive structural study has been attempted for monocyclic PCLs.^34^ With a series of macromolecular cages with varied arm numbers and molecular weights in hand, we initially investigated the weight-average hydrodynamic diameters (*D*_h_) and the weight-average intrinsic viscosities ([*η*]) in THF by employing triple-detection SEC consisting of multiangle light scattering, viscosity, and refractive index detectors (SEC-MALS-Visco). The *D*_h_ values of the macromolecular cages are in the range of 3.8–6.8 nm, as summarized in Table 1 and Fig. 4a (see also Fig. S19†). The *D*_h_ value is dependent on both the arm number and total molecular weight. Fig. 4b shows double-logarithmic plots of the *M*_W,MALS_*versus* [*η*] for the prepared linear and monocyclic PCLs as well as macromolecular cages, which clearly indicate a linear relationship between the viscosity and molecular weight. More importantly, the [*η*] values for the macromolecular cages (5.4–14.1 mg mL^–1^) are apparently lower than those of the corresponding precursors (11.1–22.0 mg mL^–1^; Tables S2–S5 and Fig. S20†), despite their comparable molecular weights. In addition, the [*η*] values of the macromolecular cages further decrease with increasing arm number. A similar trend was observed in a series of multicyclic polymers.^35^,^36^ These solution state studies demonstrate that the increase in the arm number or decrease in the molecular weight of the macromolecular cage results in a lower hydrodynamic volume, which supports the possibility of controlling the inner cavity size.

Since PCL is a typical crystalline polymer, its solid state properties are also of particular interest. Thus, the melting temperature (*T*_m_), crystallinity (*X*_WAXD_), and lamellar thickness in the PCL crystal long period were then examined by differential scanning calorimetry (DSC), wide-angle X-ray diffraction (WAXD), and small-angle X-ray scattering (SAXS) measurements, respectively. Note that a series of samples discussed in Fig. 4c and d had a *M*_n,NMR_ of *ca.* 9000 and thus their arm lengths are reduced with increasing arm number. The *T*_m_ value of the **ring-b** (56.0 °C) is found to be slightly higher than that of the linear counterpart **Pre_ring_-b** (52.4 °C). Ree and Saalwächter groups also reported a similar trend in the comparison between linear and ring PCLs.^34^,^37^ In contrast, the *T*_m_ and *X*_WAXD_ values of macromolecular cages apparently decrease with increasing arm number. For example, the *T*_m_ and *X*_WAXD_ of **cage_6_-b** are determined to be 31.8 °C and 28.2%, respectively, which are apparently lower than those of **ring-b** (56.0 °C and 38.7%, respectively) and **cage_3_-b** (52.5 °C and 38.8%, respectively). On the other hand, **cage_8_-b** is found to hardly crystallize. These results implied the distinctive difference in crystallization behaviors between the single cyclic polymer and macromolecular cages. An increase in the arm number causes a decrease in the chain mobility and chain packing ability as well as reduction in each arm length, resulting in less or no crystalline formation in the macromolecular cages (Fig. S23†).^38^,^39^ To further gain an insight into the crystallization behaviors, SAXS analysis was performed on the PCL samples, which provided information about the crystalline lamellar layer formation in crystalline-amorphous two-phase systems (Fig. S24–S28†). Based on the correlation function analysis of the SAXS profiles,^40^ we estimated the ratio of the crystalline lamellae thickness (*L*_c_) and long period (*L*_ac_), *i.e.*, *L*_c_/*L*_ac_ (Fig. 4d). The *L*_c_/*L*_ac_ values of the macromolecular cages decrease with increasing arm number, whereas no significant change is observed in their *L*_ac_ (see Tables S1–S5†). In a similar manner to the *T*_m_ and *X*_WAXD_ values, a significant decrease in the lamellae thickness (37.2–43.1% and N.D.) is observed with increasing arm number, which can also be considered due to the suppressed molecular mobility and diminished chain-packing ability. The increased *L*_c_/*L*_ac_ value in **ring-b**, as compared to its linear counterpart, seems to be correlated with its increased *T*_m_ value. It is also worth noting that the *T*_m_, *X*_WAXD_, and *L*_c_/*L*_ac_ of the macromolecular cages tend to be lower than those of the star-shaped precursors, despite their equivalent arm length. This suggests that one additional junction point to construct the cage-shaped architecture can bring about a significant impact on the solid state properties. The difference in the crystallization behaviors between the macromolecular cages and the corresponding star-shaped precursors is more pronounced when the arm number is increased. For example, low molecular weight macromolecular cages having six- and eight-arms (**cage_6_-a** and **cage_8_-a**; *M*_n,NMR_ = *ca.* 6000) do not show any evidence of crystallization from the WAXD analysis (Fig. S29–S33†), while their star-shaped precursors displayed distinct scattering peaks corresponding to the PCL crystal structure (Fig. S27, S28, S32 and S33†). Interestingly, **cage_8_-b** (*M*_n,NMR_ = 9530) with a degree of polymerization of each arm of around eight is still amorphous, despite the fact that the linear caprolactone tetramer can be crystallized.^41^ This demonstrates cage-shaped topological effects on the solid state properties.

## Conclusions

In this study, we have successfully established a robust and versatile synthetic strategy for macromolecular cages with desired arm numbers and sizes based on the intramolecular consecutive ROMO of the highly reactive norbornenyl groups attached to star-shaped polymer precursors. To the best of our knowledge, this study provides the first successful example of constructing multi-ring polymers through chain-reaction-type cyclization. In addition, we were able to systematically evaluate the polymer properties associated with the cage-shaped architecture, which revealed that the hydrodynamic diameter, viscosity, and crystallization behavior of macromolecular cages are strongly affected by the arm number and arm length. The synthetic strategy proposed in this paper should be applicable to a wide range of polymer backbones, enabling access to macromolecular cages with unique structures and functions that will open new avenues for research in supramolecular chemistry and materials science.

## Conflicts of interest

There are no conflicts to declare.

## Supplementary Material

Supplementary information
