# Supplementary material for: A versatile synthetic strategy for macromolecular cages: intramolecular consecutive cyclization of star-shaped polymers
Source: Chem Sci. 2018 Oct 11;10(2):440–6. doi: 10.1039/c8sc04006k (PMC6335864; doi:10.1039/c8sc04006k)
Supplement: Supplementary file 1 [file SC-010-C8SC04006K-s001.pdf]

Electronic Supplementary Material (ESI) for Chemical Science.
This journal is © The Royal Society of Chemistry 2018

Electronic Supplementary Information

A versatile synthetic strategy to macromolecular cages: Intramolecular consecutive cyclization of star-shaped polymers

Yoshinobu Mato,[†] Kohei Honda,[†] Kenji Tajima,[‡] Takuya Yamamoto,[‡] Takuya Isono,[‡,*] Toshifumi Satoh[‡,*]

[†]*Graduate School of Chemical Sciences and Engineering, Hokkaido University, Sapporo 060-8628, Japan*

[‡]*Division of Applied Chemistry, Faculty of Engineering, Hokkaido University, Sapporo 060-8628, Japan*

Table of Contents

S1. Experimental Section 3

S1-1. Materials 3

S1-2. Instruments 4

S1-3. Synthetic details 7

S2. Additional Results 43

S2-1. Systematic evaluation of physical properties for macromolecular cages and their precursors 43

S2-2. SAXS experiment 46

S2-3. WAXD experiment 49

S2-4. Long period estimation by correlation function analysis 54

S3. References 55

S1. Experimental Section

S1-1. Materials.

Grubbs' catalyst 3rd generation (G3)[1] and 5-(hydroxymethyl)-2,2,5-trimethyl-1,3-dioxane (HTMD)[2] were prepared according to previously reported method. Amberlyst® A21 (Organo Co., Ltd), *N*,*N*-dimethyl-4-aminopyridine (DMAP; Tokyo Chemical Industry Co., Ltd. (TCI), >99.0%), 1-ethyl-3-(3-(dimethylamino)-propyl)carbodiimide hydrochloride (EDC; TCI, >98.0%), ethyl vinyl ether (TCI, >98.0%), (±)-*exo*-5-norbornenecarboxylic acid (*exo*-NB-COOH; Aldrich, 97%), sodium iodide (NaI; Wako Pure Chemical Industry Co. Ltd., >99.5%), and 2, 5-dihydroxybenzoic acid (DHB; Sigma-Aldrich, >98.0%) were used as received. *ε*-Caprolactone (*ε*-CL; TCI, >99%) was purified by distillation over CaH_2 under reduced pressure and stored in the glovebox. Diphenyl phosphate (DPP; TCI, >99.0%), 2,2-diethyl-1,3-propanol (TCI, >98.0%), trimethylolpropane (TCI, >98.0%), pentaerythritol (TCI, >98.0%), and dipentaerythritol (TCI, >98.0%) were purified by azeotropic distillation with dry toluene and stored in the glovebox.

S1-2. Instruments.

The polymerization experiments were carried out in an MBRAUN stainless steel glovebox equipped with a gas purification system (molecular sieves and copper catalyst) in a dry argon atmosphere (H_2O, O_2<0.1 ppm). The moisture and oxygen contents in the glovebox were monitored by an MB-MO-SE 1 moisture sensor and an MB-OX-SE 1 oxygen sensor, respectively. Dry toluene (>99.5%; water content, <0.001%, Kanto Chemical Co., Inc.) used for the polymerization was purified by passing through an MBRAUN solvent purification system (MB SPS COMPACT) consisting of a column of activated alumina and a column with activated copper catalyst. The ^{1}H (400 MHz) and ^{13}C NMR (100 MHz) spectra were recorded using a JEOL JNM-ECS400 instrument at room temperature in $CDCl_3$ or methanol-d_4. The size exclusion chromatography (SEC) was performed at 40 °C in THF (flow rate, 1.0 mL min^{-1}) using a Shodex GPC-101 gel permeation chromatography system (Shodex DU-2130 dual pump, Shodex RI-71-S reflective index detector, and Shodex ERC-3125SN degasser) equipped with a Shodex KF-G guard column (4.6 mm × 10 mm; particle size, 8 μm) and two Shodex KF-804L columns (linear, 8 mm × 300 mm). The number-average molecular weight ($M_{n,SEC}$) and the dispersity ($Đ$) of the polymers were calculated on the basis of polystyrene calibrations. The absolute molecular weights ($M_{w,MALS}$) of the samples of macromolecular cages and their star-shaped polymer precursors were determined by SEC with multiangle light scattering detection (SEC-MALS-Visco) in THF (flow rate, 1.0 mL min^{-1}) at 40 °C using an Agilent 1100 series instrument equipped with a DG 1100 degasser, a Shodex KF-G guard column (4.6 mm × 10 mm; particle size, 8 μm), a Shodex KF-

800D solvent-peak separation column (linear, 8.0 mm × 100 mm; particle size, 10μm), two Shodex KF-805L columns (linear, 8.0 mm × 300 mm; exclusion limit, 4.0 × 10^6; particle size, 10 μm), a DAWN 8+ multiangle laser light scattering detector (Wyatt Technology), an Optilab rEX refractive index detector (Wyatt Technology), and a Viscostar viscosity detector (Wyatt Technology). The preparative SEC for Grubbs' catalyst removal was performed at r.t. in CH_3Cl (flow rate, 3.5 mL min^{-1}) using LC-9201 liquid chromatography system (Japan Analytical Industry Co. Ltd.) equipped with a BG-12 degasser, a PI-50 pump, a RI-50S RI detector, a JAIGEL-H-P guard column (8 mm × 40 mm; Japan Analytical Industry Co. Ltd.), and a Shodex K-2004 column (linear, 20.0 mm × 300 mm; exclusion limit, 1.4 × 10^4; particle size, 7 μm). The matrix-assisted laser desorption ionization time-of-flight mass spectrometry (MALDI-TOF MS) of the obtained polymers was performed using an Applied Biosystems Voyager-DE STR-H equipped with a 337 nm nitrogen laser (3 ns pulse width). Two hundred shots were accumulated for the spectra at a 20 kV acceleration voltage in the reflector mode and calibrated using PSt as the internal standard. Samples for the MALDI-TOF MS were prepared as follows: (i) the polymer sample (1.0 mg) and DHB (2.0 mg) as a matrix were dissolved in THF (3.0 mL). (ii) Then, the solution was added to the THF solution of cationic agent (NaI; 1.0 mg mL^{-1}, 1.0 mL). (iii) The mixed solution was dropped to a sample plate. The thermal properties of the polymer samples were measured from −50 to 100 °C during the second heating by a Bruker AXS DSC 3100 differential scanning calorimeter under a nitrogen atmosphere with the heating rate of 10 °C min^{-1} and cooling rate 20 °C min^{-1}. Synchrotron small-angle X-ray scattering (SAXS) and wide-angle

X-ray diffraction (WAXD) measurements of the obtained macromolecular cages and their precursors were performed with an X-ray beam of 1.5Å at the BL-6A in the Photon Factory (Tsukuba, Japan). The 2D SAXS and WAXD profiles were obtained with a Pilatus 1M and 100K detectors, respectively, which were circularly averaged to produce the 1D plots of log *I* (intensity) and *q* (scattering vector). The *q* value was calibrated using a silver behenate. The powder sample of the polymer was put into a Hilgenberg lindemann glass capillary (1.5 mm × 80 mm), which was annealed at 100 °C for 1 h in a pre-heated oven to erase a thermal history and then cooled to room temperature.

S1-3. Synthetic Details

General synthetic procedure for the polymer precursors

A linear PCL with a reactive *exo*-norbornenes at each chain end (**Pre$_{ring}$-a**) was prepared in two steps consisting of polymerization of ε-CL and subsequent end-functionalization with *exo*-norbornene carboxylic acid (*exo*-NB-COOH). The DPP-catalyzed ring-opening polymerization of ε-CL was carried out using 2,2-diethyl-1,3-propanediol as an initiator with the [ε-CL]$_0$/[I]$_0$/[DPP] ratio of 50/1/0.05 to afford linear PCL (**HO-PCL-OH-a**; $M_{n,NMR}$ = 4,890, $Đ$ = 1.05). Subsequently, **HO-PCL-OH-a** was treated with *exo*-NB-COOH in the presence of EDC and DMAP ([**HO-PCL-OH-a**]$_0$/[*exo*-NB-COOH]$_0$/[DMAP]/[EDC] = 1/4/6/6) to generate **Pre$_{ring}$-a**. In the ^{1}H NMR spectrum of **Pre$_{ring}$-a**, a proton signal attributed to the methylene adjacent to each chain end (*i*: 3.65 ppm) were observed. After the subsequent condensation reaction with *exo*-NB-COOH, the ^{1}H NMR signals due to the norbornenyl groups (*x, y*: 6.13 ppm; *z*: 3.04 ppm; *w*: 2.93 ppm; *v*: 2.22 ppm; *u*: 1.91 ppm) appeared and suggested quantitative introduction of the norbornenyl group to the chain ends. In addition, the SEC traces of **Pre$_3$-a** retained the monomodal peak with $Đ$ = 1.05. These results supported the successful synthesis of **Pre$_3$-a** ($M_{n,NMR}$ = 5,110, $M_{n,SEC}$ = 9,790, $Đ$ = 1.05).

Synthesis of liner PCL (HO-PCL-OH)

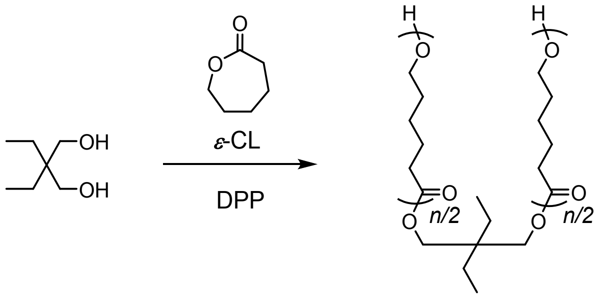

A typical procedure for the polymerization is as follows (method A): Under Ar atmosphere, ε-CL (1.50 g, 13.1 mmol), 2,2-diethyl-1,3-propanediol (21.7 mg, 164 μmol), and DPP (33.7 mg, 164 μmol) were placed in a reaction vessel. The reaction mixture was stirred at 80 °C for 2.5 h. The polymerization was quenched by the addition of Amberlyst® A21. The polymer crude was purified by the reprecipitation from CH_2Cl_2 into cold methanol/*n*-hexane (v/v = 10/1) to give HO-PCL-OH-a as a white solid. Yield: 65.3%. ^{1}H NMR (400 MHz, $CDCl_3$): δ (ppm) 4.27-3.96 (m, $(CH_3CH_2)_2CCH_2$, -$OCO(CH_2)_4CH_2$–), 3.91 (s, $(CH_3CH_2)_2C$–), 3.65 (q, J = 6.1, –CH_2OH), 2.52-2.16 (m, –$OCOCH_2(CH_2)_4$–), 1.83-1.53 (m, –$OCOCH_2CH_2(CH_2)_3$–, –$OCO(CH_2)_3CH_2CH_2$–), 1.48-1.21 (m, CH_3CH_2–, –$OCO(CH_2)_2CH_2(CH_2)_2$–), 0.82 (t, J = 7.5, CH_3CH_2–). $M_{n,NMR}$ = 4,890 g mol^{-1} ($CDCl_3$), $M_{n,SEC}$ = 9,600 g mol^{-1} (THF), $Đ$ = 1.05.

Synthesis of ω-norbornenyl end-functionalized linear PCL (Pre$_{ring}$)

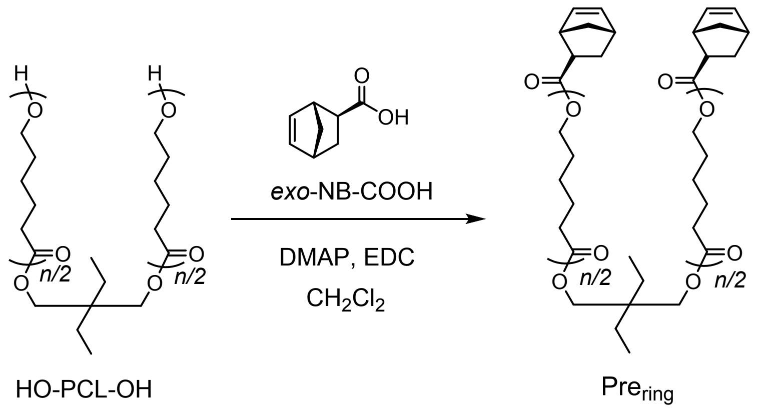

A typical procedure for the condensation reaction is as follows (method B): In a Schlenk flask, HO-PCL-OH ($M_{n,NMR}$ = 4,890 g mol^{-1}, 1.10 g, 224 μmol), *exo*-NB-COOH (78.7 mg, 570 μmol), DMAP (104 mg, 855 μmol), and EDC (221 mg, 855 μmol) were dissolved in CH_2Cl_2 (10 mL) and the mixture was stirred at r.t. for 24 h. The polymer crude was purified by reprecipitation from CH_2Cl_2 into cold methanol to give **Pre$_{ring}$-a** as a white solid. Yield: 62.2%. ^{1}H NMR (400 MHz, $CDCl_3$): δ (ppm) 6.13 (m, –C*H*=C*H*– in norbornene ring), 4.28-3.98 (m, $CH_3CH_2CCH_2$-, –$OCO(CH_2)_4CH_2$–), 3.92 (s, $(CH_3CH_2)_2C$–), 3.04 (s, –C*H*-CH-CH_2O– in norbornene ring), 2.93 (s, –C*H*–CH_2–CH–CH_2O– in norbornene ring), 2.51-2.16 (m, –$OCOCH_2(CH_2)_4$–), 1.91 (m, *exo*-C*H*– of –CH–CH_2–CH–CH_2O– in norbornene ring), 1.77-1.54 (m, –$OCOCH_2CH_2(CH_2)_3$–, –$OCO(CH_2)_3CH_2CH_2$–), 1.49-1.24 (m, CH_3CH_2–, –$OCO(CH_2)_2CH_2(CH_2)_2$–, bridge head –CH_2– in norbornene ring, *endo*-C*H*- of –CH–CH_2–CH–CH_2O–), 0.92-0.79 (t, J =7.5, CH_3CH_2–). $M_{n,NMR}$ = 5,110 g mol^{-1} ($CDCl_3$), $M_{n,SEC}$ = 9,790 g mol^{-1} (THF), $Đ$ = 1.05.

Synthesis of cyclic PCL (ring)

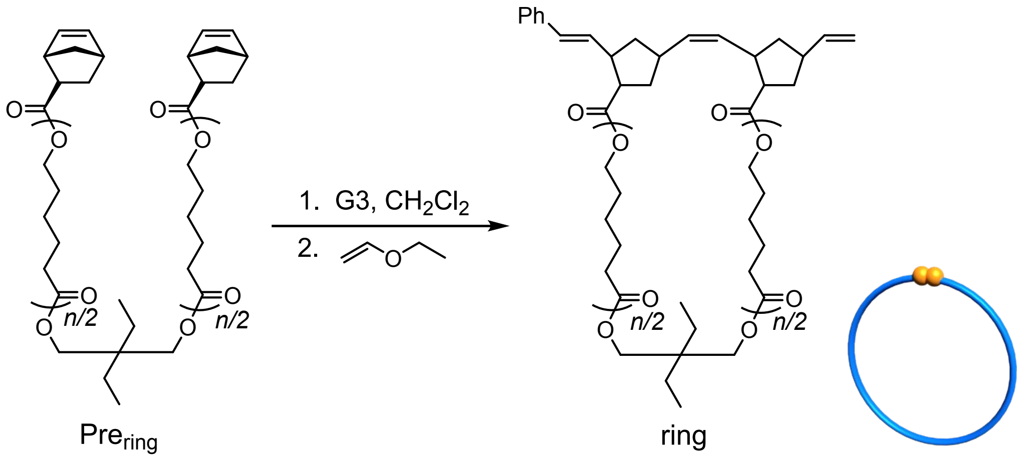

A typical procedure for the intramolecular consecutive cyclization is as follows (method C); G3 (31.2 mg, 35.2 μmol) was added to a three-necked flask and dissolved in deggased-CH_2Cl_2 (230 mL). Then, a solution of **Pre$_{\mathbf{ring}}$-a** ($M_{n,NMR}$ = 5,110 g mol^{-1}, 30.0 mg, 5.87 μmol, 170 μM in CH_2Cl_2) was added dropwise to the G3 solution through the additional funnel over 30 min. After 10 min, the reaction was quenched by the addition of ethyl vinyl ether (300 μL). The metal residue in the crude product was removed by preparative SEC (solvent, CH_3Cl) to give **ring-a** as a pale brown solid. Yield: 92.3%. ^{1}H NMR (400 MHz, $CDCl_3$): δ (ppm) 7.70, 7.53 (Aromatic), 6.59-4.82 (br, alkenyl of poly(norbornene) backbone), 3.91 (s, $(CH_3C\mathit{H}_2)_2C$–), 2.39-2.22 (m, –$OCOC\mathit{H}_2(CH_2)_4$–), 3.49-1.12 (br, cyclopentane ring of poly(norbornene) backbone), 1.77-1.58 (m, -$OCOCH_2C\mathit{H}_2(CH_2)_3$-, -$OCO(CH_2)_3C\mathit{H}_2CH_2$-), 1.47-1.29 (m, $CH_3C\mathit{H}_2$–, –$OCO(CH_2)_2C\mathit{H}_2(CH_2)_2$–), 0.83 (t, J = 7.5, $C\mathit{H}_3CH_2$–). $M_{n,SEC}$ = 7,520 g mol^{-1} (THF), $Đ$ = 1.09.

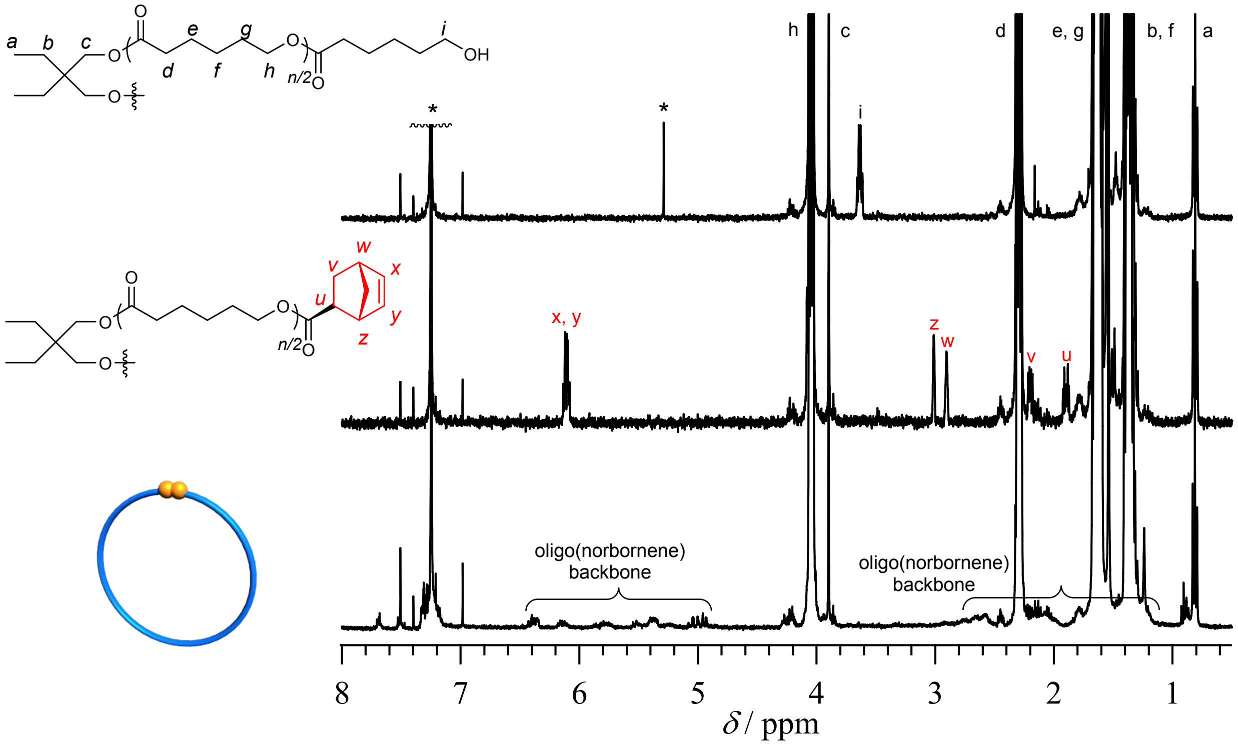

Figure S1. ^{1}H NMR spectra of **HO-PCL-OH-a** ($M_{n,NMR}$ = 4,890, $Đ$ = 1.06; upper), **Pre$_{ring}$-a** ($M_{n,NMR}$ = 5,510, $Đ$ =1.05; middle), and **ring-a** ($Đ$ = 1.09; lower) in $CDCl_3$. Asterisks show solvent signals.

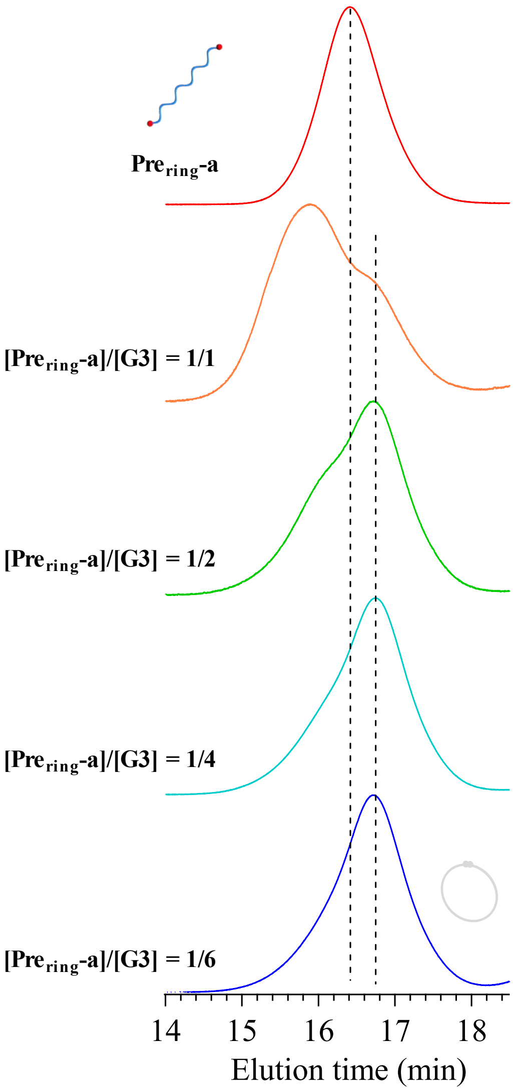

Figure S2. SEC traces of linear polymer precursor (**Pre$_{ring}$-a**) and ROMO product under various [**Pre$_{ring}$-a**]/[G3] ratio (RI detection; eluent, THF; flow rate, 1.0 mL min^{-1}).

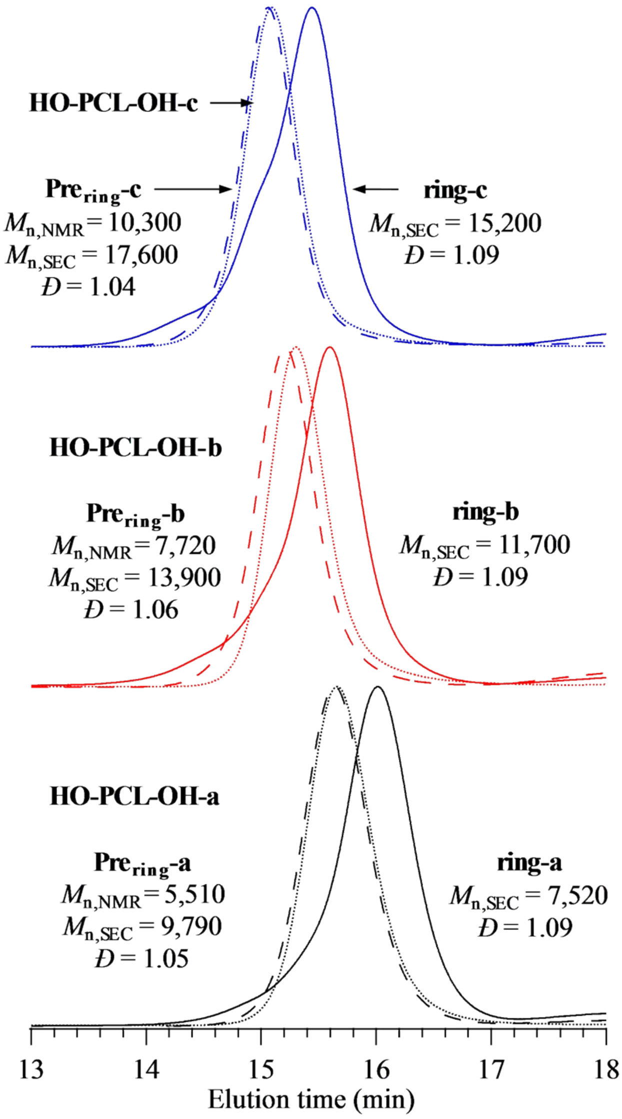

Figure S3. SEC traces of **HO-PCL-OH**s (dashed line), **Pre$_{ring}$**s (dotted line), and **ring**s (solid line) with different molecular weight.

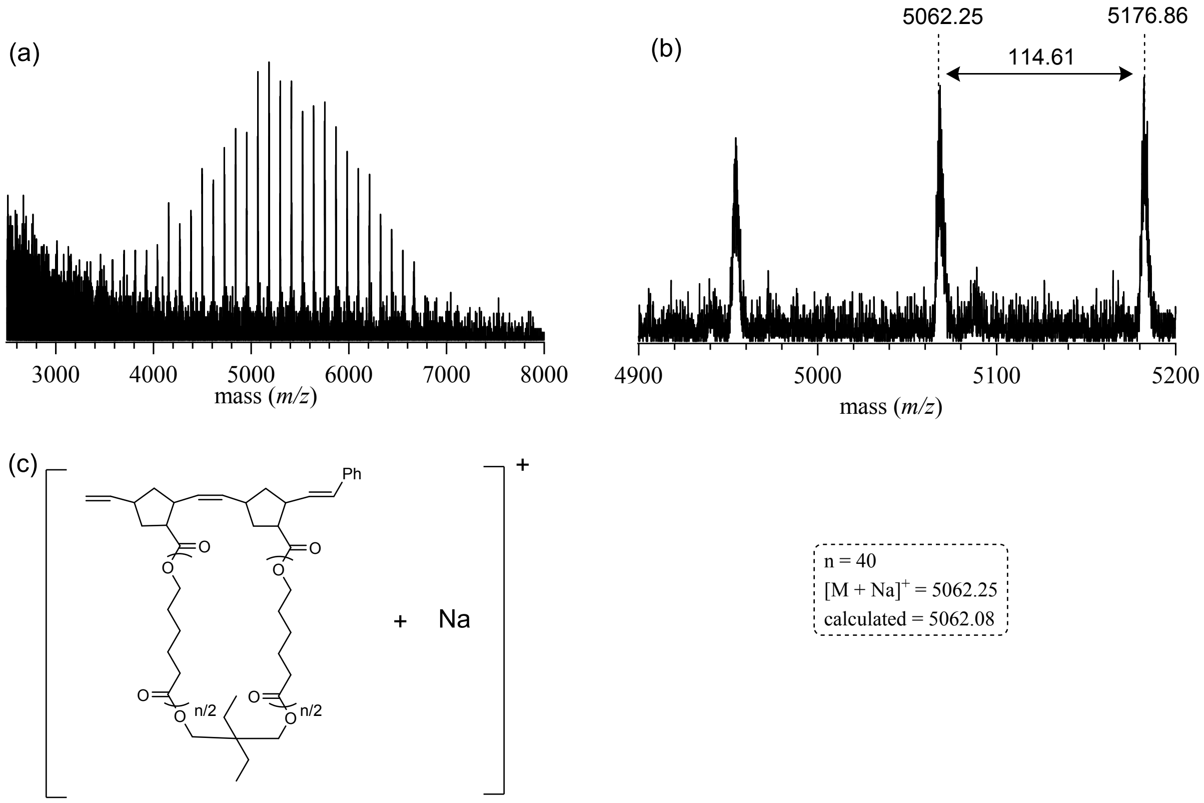

Figure S4. MALDI-TOF MS analysis of the obtained **ring-a**.

Table S1. Molecular characterization of cyclic PCLs (**ring**) and their precursors

Sample	$M_{n,NMR}$ [a]	$M_{n,SEC}$ [b]	$M_{w,MALS}$ [c]	$Đ$ [b]	yield (%)	D_h [c] (nm)	$[\eta]$ [c] (mL g^{-1})	T_m [d] (°C)	X_{WAXD} [e] (%)	L_{ac} [f] (nm)	L_c/L_{ac} [f] (%)
HO-PCL-OH-a	4,890	9,620	-	1.06	65.3	-	-	-	-	-	-
Pre$_{ring}$-a	5,510	9,790	5,590	1.05	61.0	5.2	17.5	43.2	35.2	11.1	39.7
ring-a	-	7,520	6,280	1.09	92.3	4.6	11.3	51.1	40.3	11.0	38.5
HO-PCL-OH-b	7,480	13,400	-	1.05	73.3	-	-	-	-	-	-
Pre$_{ring}$-b	7,720	13,900	8,040	1.06	72.6	6.4	22.4	52.4	38.8	11.1	41.7
ring-b	-	11,700	8,900	1.09	92.3	5.8	15.8	56.0	38.7	11.0	43.1
HO-PCL-OH-c	10,800	17,300	-	1.05	86.0	-	-	-	-	-	-
Pre$_{ring}$-c	10,300	17,600	11,500	1.04	87.0	7.4	27.7	53.6	35.9	11.2	40.4
ring-c	-	15,200	11,600	1.07	91.6	6.8	18.8	56.9	42.5	N.D.[g]	N.D.[g]

[a] Determined by ^{1}H NMR. [b] Determined by SEC in THF using PSt standards. [c] Determined by SEC-MALS-Visco in THF. $D_h = 2\ R_h = 2(3V_h/4\pi)^{1/3}$; V_h is hydrodynamic volume. V_h was calculated by Einstein–Simha equation ($V_h = M_{w,MALS}[\eta]/2.5N_A$; N_A: Avogadro's number). [d] Determined from a melting peak of the DSC curve. [e] Determined by WAXD at r.t. [f] Determined by SAXS at r.t. [g] Not determined.

Synthesis of three-armed star-shaped PCL (*s*-(PCL-OH)$_3$)

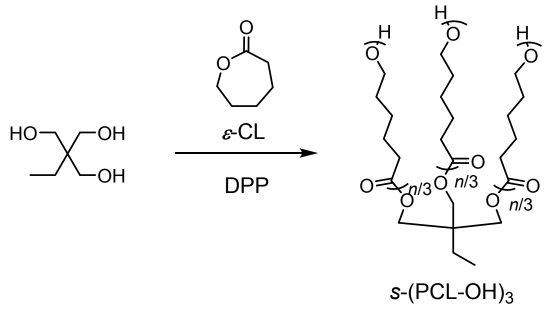

Method A was used for the polymerization of *ε*-CL (3.00 g, 26.3 mmol) with trimethylolpropane (70.0 mg, 526 μmol) and DPP (6.6 mg, 26 μmol) at 80 °C for 2.5 h to give ***s*-(PCL-OH)$_3$-a** as a white solid. Yield: 77.5%. ^{1}H NMR (400 MHz, CDCl$_3$): δ (ppm) 3.98-4.09 (m, CH$_3$CH$_2$CC*H*$_2$–, –OCO(CH$_2$)$_4$C*H*$_2$–), 3.63 (q, *J* = 6.00, -C*H*$_2$OH), 2.30 (m, –OCOC*H*$_2$(CH$_2$)$_4$–), 1.55-1.68 (m, –OCOCH$_2$C*H*$_2$(CH$_2$)$_3$–, –OCO(CH$_2$)$_3$C*H*$_2$CH$_2$–), 1.32-1.44 (m, CH$_3$C*H*$_2$–, –OCO(CH$_2$)$_2$C*H*$_2$(CH$_2$)$_2$–), 0.87 (t, *J* = 15.2, C*H*$_3$CH$_2$–). $M_{n,NMR}$ = 5,530 g mol^{-1} (CDCl$_3$), $M_{n,SEC}$ = 9,440 g mol^{-1} (THF), $Đ$ = 1.07.

Synthesis of *ω*-norbornenyl end-functionalized three-armed star-shaped PCL (Pre_3)

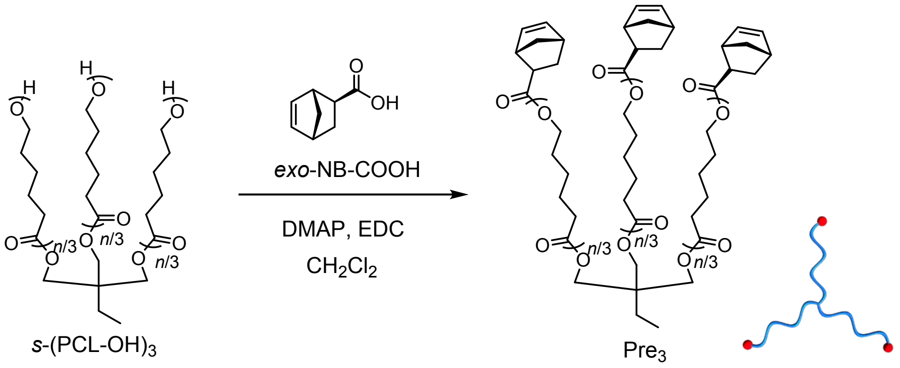

Method B was used for the reaction of ***s*-(PCL-OH)₃-a** ($M_{n,NMR}$ = 5,530, 1.00 g, 181 μmol) with *exo*-NB-COOH (150 mg, 1.08 mmol) in the presence of DMAP (199 mg, 1.66 μmol) and EDC (312 mg, 1.66 mmol) in CH_2Cl_2 (10 mL) to give **Pre_3-a** as a white solid. Yield: 58.6%. ^{1}H NMR (400 MHz, $CDCl_3$): δ (ppm) 6.11 (m, –C*H*=C*H*– in norbornene ring), 4.08-3.97 (m, $CH_3CH_2CCH_2$–, –OCO$(CH_2)_4CH_2$–), 3.01 (s, –C*H*–CH–CH_2O– in norbornene ring), 2.90 (s, –C*H*–CH_2–CH–CH_2O– in norbornene ring), 2.29 (m, –OCOC$H_2(CH_2)_4$–), 2.20 (m, –CH–CH_2–C*H*–CH2O– in norbornene ring), 1.89 (m, *exo*–C*H*– of CH–CH_2–CH–CH_2O– in norbornene ring), 1.58-1.66 (m, –OCO$CH_2CH_2(CH_2)_3$–, –OCO$(CH_2)_3CH_2CH_2$–), 1.33-1.41 (m, CH_3CH_2-, –OCO$(CH_2)_2CH_2(CH_2)_2$–, bridge head –CH_2– in norbornene ring, *endo*-C*H*– of –CH–CH_2–CH–CH_2O–), 0.87 (t, J = 15.2, CH_3CH_2–). $M_{n,NMR}$ = 6,000 g mol^{-1} ($CDCl_3$), $M_{n,SEC}$ = 9,810 g mol^{-1} (THF), $Đ$ = 1.05.

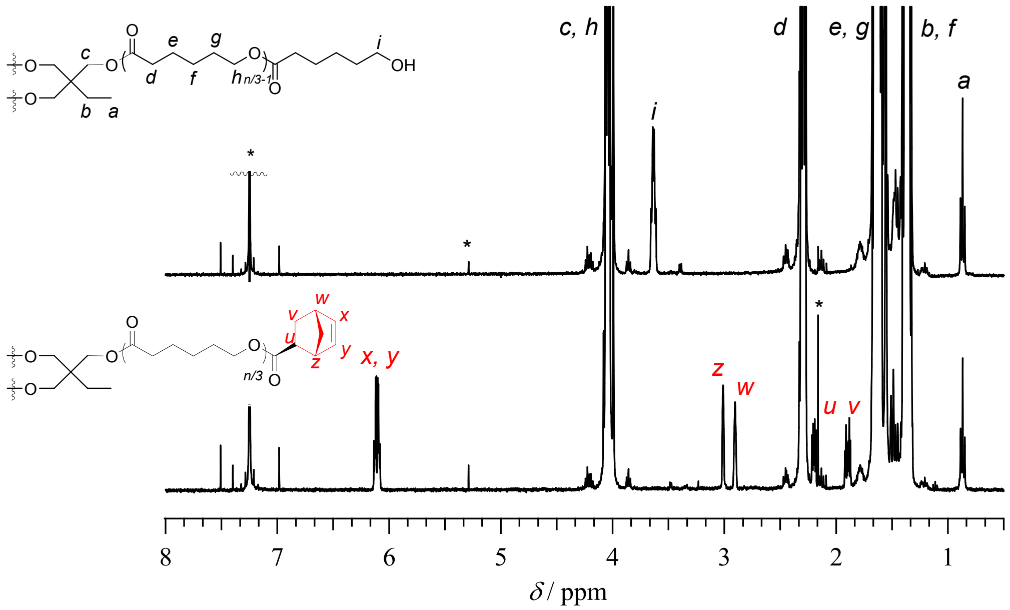

Figure S5. ^{1}H NMR spectra of *s*-(PCL-OH)$_3$-a ($M_{n,NMR}$ = 5,530, $Đ$ = 1.07; upper) and **Pre$_3$-a** ($M_{n,NMR}$ = 6,000, $Đ$ = 1.05; lower) in $CDCl_3$. Asterisks show solvent signals.

Synthesis of three-armed macromolecular cage ($cage_3$) via intramolecular ROMO

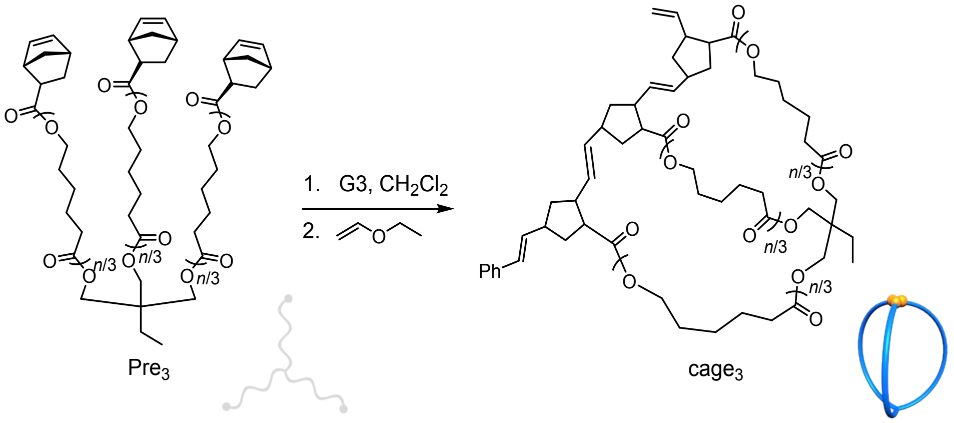

Method C was used for the ROMO of **Pre_3-a** ($M_{n,NMR}$ = 6,000 g mol^{-1}, 30.0 mg, 5.00 µmol, 170 µM in CH_2Cl_2) with G3 (26.6 mg, 30.0 µmol) in CH_2Cl_2 (230 mL) to give **$cage_3$-a** as a pale brown solid. Yield: 92.3%. ^{1}H NMR (400 MHz, $CDCl_3$): δ (ppm) 7.70, 7.54 (Aromatic), 7.70-4.80 (br, alkenyl of poly(norbornene) backbone), 4.08-3.97 (m, $CH_3CH_2CCH_2$–, –OCO$(CH_2)_4CH_2$–), 2.79-1.10 (br, cyclopentane ring of poly(norbornene) backbone), 2.29 (m, –OCO$CH_2(CH_2)_4$–), 1.66-1.58 (m, –OCO$CH_2CH_2(CH_2)_3$–, –OCO$(CH_2)_3CH_2CH_2$–), 1.41-1.33 (m, CH_3CH_2–, –OCO$(CH_2)_2CH_2(CH_2)_2$–), 0.87 (t, J = 15.2, CH_3CH_2–). $M_{n,SEC}$ = 6,770 g mol^{-1} (THF), $Đ$ = 1.09.

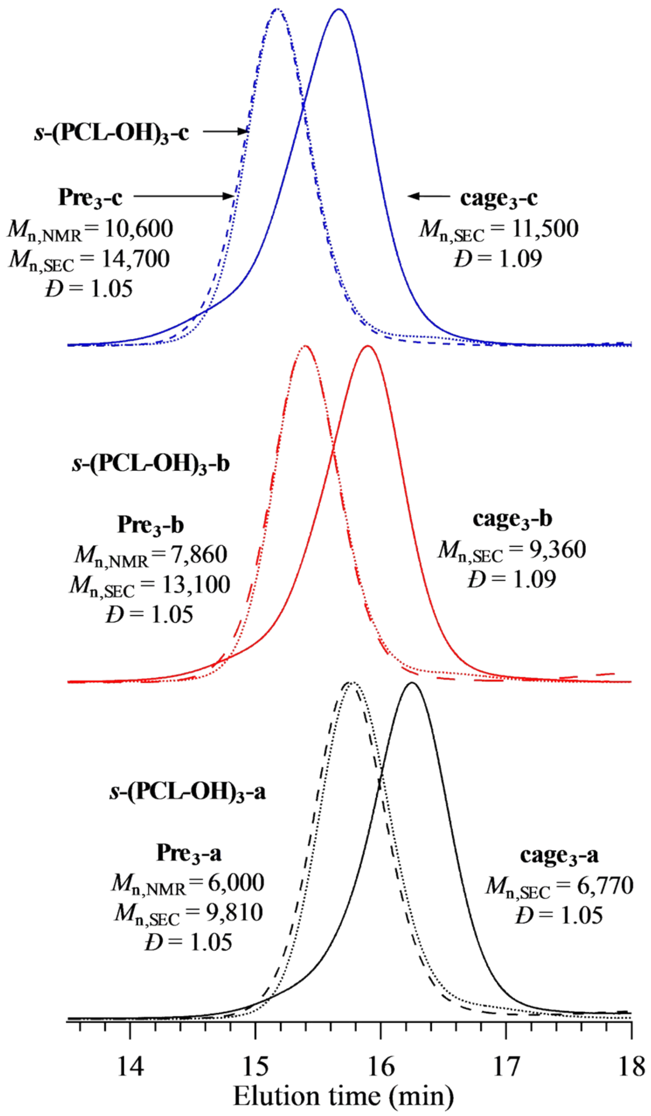

Figure S6. SEC traces of ***s*-(PCL-OH)$_3$**s (dashed line), **Pre$_3$**s (dotted line), and **cage$_3$**s (solid line) with different molecular weight.

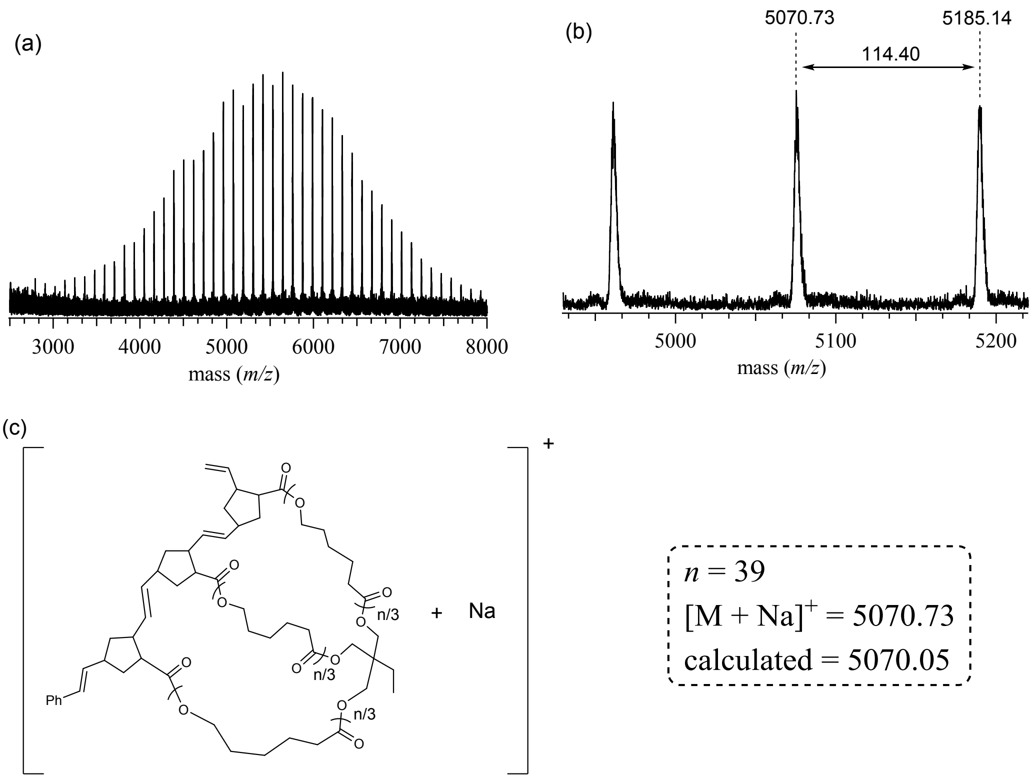

Figure S7. MALDI-TOF MS analysis of the obtained **cage$_3$-a**.

Table S2. Molecular characterization of three-armed macromolecular cages (**cage$_3$**) and their precursors

Sample	$M_{n,NMR}$ ^{a}	$M_{n,SEC}$ ^{b}	$M_{w,MALS}$ ^{c}	$Đ$ ^{b}	yield (%)	D_h ^{c} (nm)	$[\eta]$ ^{c} (mL g^{-1})	T_m ^{d} (°C)	X_{WAXD} ^{e} (%)	L_{ac} ^{f} (nm)	L_c/L_{ac} ^{f} (%)
s-(PCL-OH)$_3$-a	5,530	9,440	-	1.07	77.5	-	-	-	-	-	-
Pre$_3$-a	6,000	9,810	5,900	1.05	63.2	4.8	16.3	44.5	36.8	12.3	37.1
cage$_3$-a	-	6,770	7,550	1.09	92.3	4.4	9.1	46.3	33.9	10.8	38.2
s-(PCL-OH)$_3$-b	7,730	12,100	-	1.07	82.2	-	-	-	-	-	-
Pre$_3$-b	7,860	13,100	8,060	1.05	79.5	6.0	19.6	38.47.9	39.5	12.5	38.5
cage$_3$-b	-	9,360	9,370	1.09	80.0	5.0	11.8	52.5	38.8	11.2	39.9
s-(PCL-OH)$_3$-c	9,390	14,600	-	1.06	75.6	-	-	-	-	-	-
Pre$_3$-c	10,600	14,700	9,680	1.05	39.0	6.8	22.0	49.9	38.7	11.2	40.0
cage$_3$-c	-	11,500	10,200	1.09	84.0	5.6	14.1	54.3	40.6	11.4	40.3

^{a} Determined by ^{1}H NMR. ^{b} Determined by SEC in THF using PSt standards. ^{c} Determined by SEC-MALS-Visco in THF. $D_h = 2\ R_h = 2(3V_h/4\pi)^{1/3}$; V_h is hydrodynamic volume. V_h was calculated by Einstein–Simha equation ($V_h = M_{w,MALS}[\eta]/2.5N_A$; N_A: Avogadro's number). ^{d} Determined from a melting peak of the DSC curve. ^{e} Determined by WAXD at r.t. ^{f} Determined by SAXS at r.t.

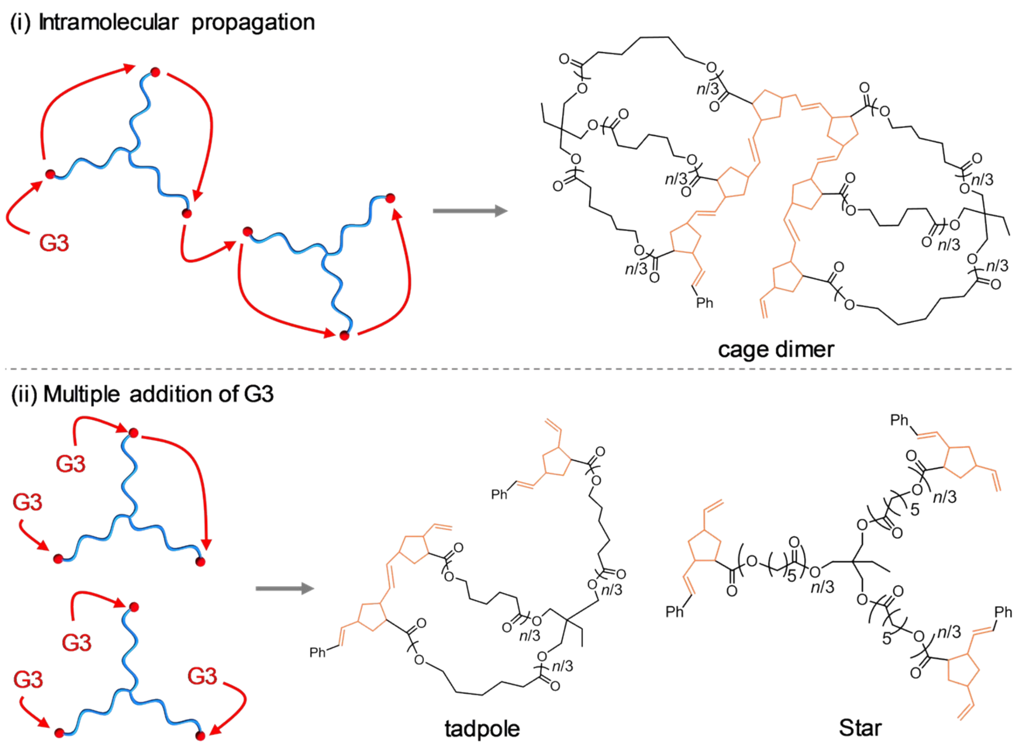

Figure S8. Schematic illustration of possible side reactions during the synthesis of **cage3-a**: (i) oligomer formation through intermolecular propagation and (ii) acyclic by-product formation through multiple addition of G3.

Synthesis of four-armed star-shaped PCL (*s*-(PCL-OH)$_4$)

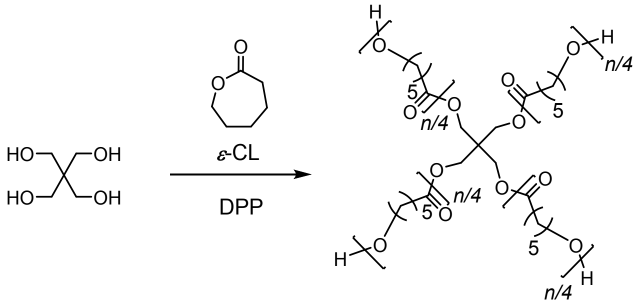

***s*-(PCL-OH)$_4$**

Method A was used for the polymerization of ε-CL (3.00 g, 26.3 mmol), pentaerythritol (70.0 mg, 526 μmol) and DPP (6.6 mg, 26 μmol) at 80 °C for 1.8 h to give ***s*-(PCL-OH)$_4$-a** as a white solid. Yield: 82.9%. ^{1}H NMR (400 MHz, $CDCl_3$): δ (ppm) 4.33-3.82 (m, $-OCO(CH_2)_4CH_2-$), 3.72-3.56 (m, $-CH_2OH$), 2.54-2.08 ($-OCOCH_2(CH_2)_4-$), 1.86-1.53 ($-OCOCH_2CH_2(CH_2)_3$-, $-OCO(CH_2)_3CH_2CH_2-$), 1.50-1.17 (m, $-OCO(CH_2)_2CH_2(CH_2)_2-$). $M_{n,NMR}$ = 5,970 g mol^{-1} ($CDCl_3$), $M_{n,SEC}$ = 9,900 g mol^{-1} (THF), $Đ$ = 1.06.

Synthesis of ω-norbornenyl end-functionalized four-armed star-shaped PCL (Pre_4)

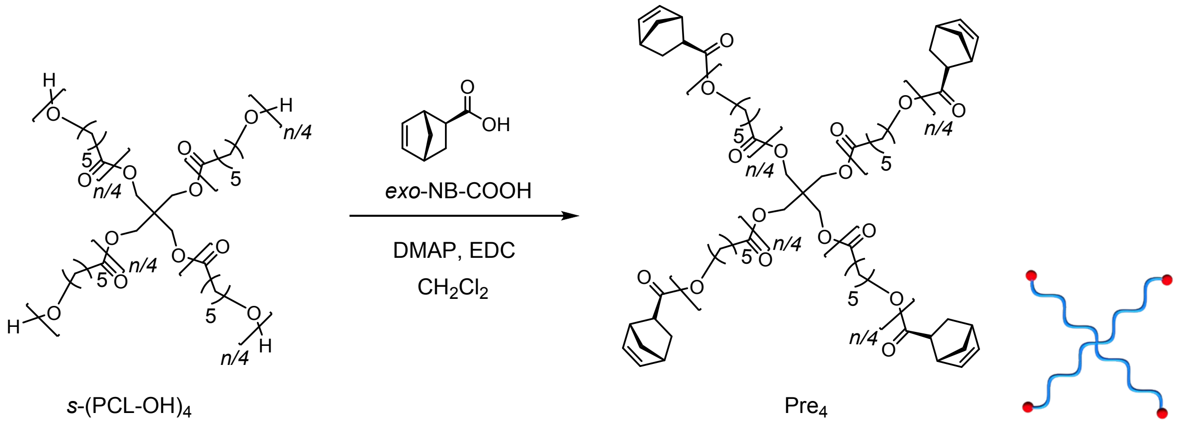

Method B was used for the reaction of ***s*-(PCL-OH)$_4$-a** ($M_{n,NMR}$ = 5,970 g mol^{-1}, 1.00 g, 168 μmol) with *exo*-NB-COOH (185 mg, 1.34 mmol) in the presence of DMAP (245 mg, 2.01 μmol), EDC (385 mg, 2.01 mmol) in CH_2Cl_2 (10 mL) to give **Pre$_4$-a** as a white solid. Yield: 66.0%. ^{1}H NMR (400 MHz, $CDCl_3$): δ (ppm) 6.12 (m, –C*H*=C*H*– in norbornene ring), 4.31-3.82 (m, –OCO(CH_2)$_4$C*H*$_2$–), 3.02 (s, –C*H*–CH–CH_2O– in norbornene ring), 2.92 (s, –C*H*–CH_2–CH–CH_2O– in norbornene ring), 2.54-2.08 (m, –OCOC*H*$_2$(CH_2)$_4$–), 1.96-1.87 (m, –OCO(CH_2)$_2$C*H*$_2$(CH_2)$_2$–, bridge head –CH_2– in norbornene ring, *endo*-C*H*– of –CH–C*H*$_2$–CH–CH_2O–), 1.74-1.47 (m, –OCOCH$_2$C*H*$_2$(CH_2)$_3$–, –OCO(CH_2)$_3$C*H*$_2$CH$_2$–), 1.46-1.22 (m, –OCO(CH_2)$_2$C*H*$_2$(CH_2)$_2$–). $M_{n,NMR}$ = 5,990 g mol^{-1} ($CDCl_3$), $M_{n,SEC}$ = 9,100 g mol^{-1} (THF), $Đ$ = 1.05.

Synthesis of four-armed macromolecular cage (cage$_4$) via intramolecular ROMO

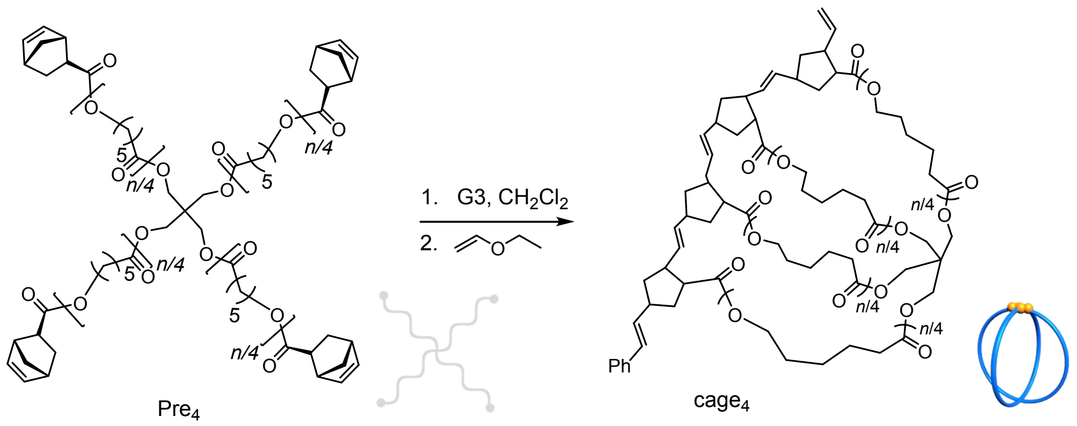

Method C was used for the ROMO of **Pre$_4$-a** ($M_{n,NMR}$ = 5,990 g mol^{-1}, 30.0 mg, 5.01 µmol, 170 µM in CH_2Cl_2) with G3 (26.6 mg, 30.0 µmol) in CH_2Cl_2 (230 mL) to give **cage$_4$-a** as a pale brown solid. Yield: 85.0%. ^{1}H NMR (400 MHz, $CDCl_3$): δ (ppm) 7.70, 7.54 (Aromatic), 6.51-4.90 (br, alkenyl of oligo(norbornene) backbone), 4.29-3.86 (m, –OCO(CH_2)$_4$CH_2–), 3.20-1.83 (br, cyclopentane ring of oligo(norbornene) backbone), 2.43-2.20 (m, –OCOCH_2(CH_2)$_4$–), 1.82-1.49 (m, –OCOCH$_2$CH_2(CH_2)$_3$–, –OCO(CH_2)$_3$CH_2CH$_2$–), 1.45-1.19 (m, –OCO(CH_2)$_2$CH_2(CH_2)$_2$–). $M_{n,SEC}$ = 6,290 g mol^{-1} (THF), $Đ$ = 1.08.

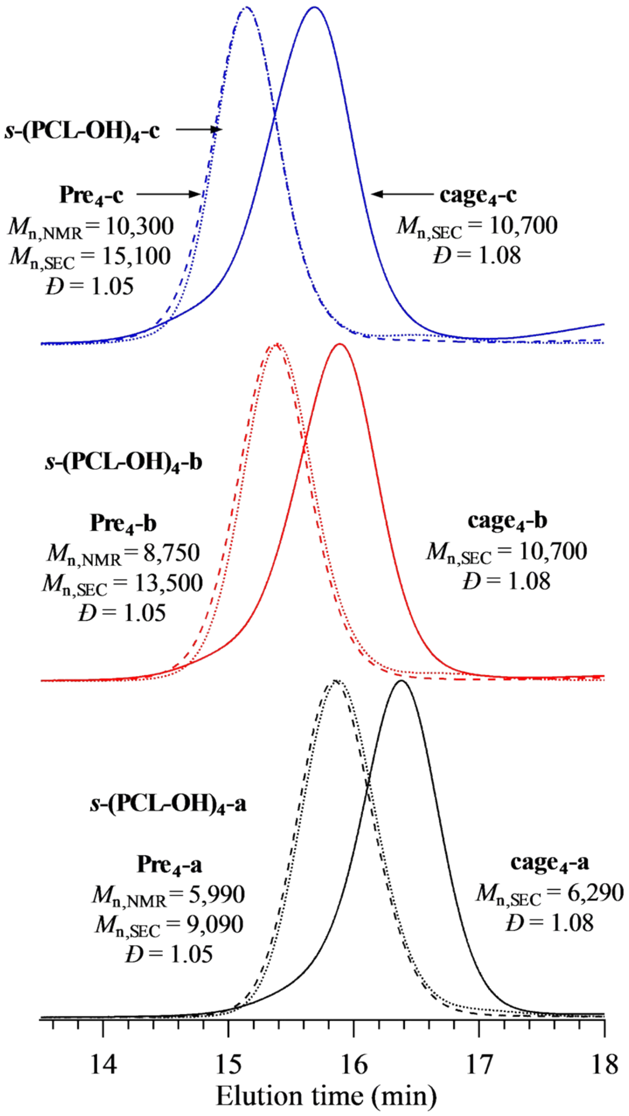

Figure S9. SEC traces of ***s*-(PCL-OH)$_4$**s (dashed line), **Pre$_4$**s (dotted line), and **cage$_4$**s (solid line) with different molecular weight.

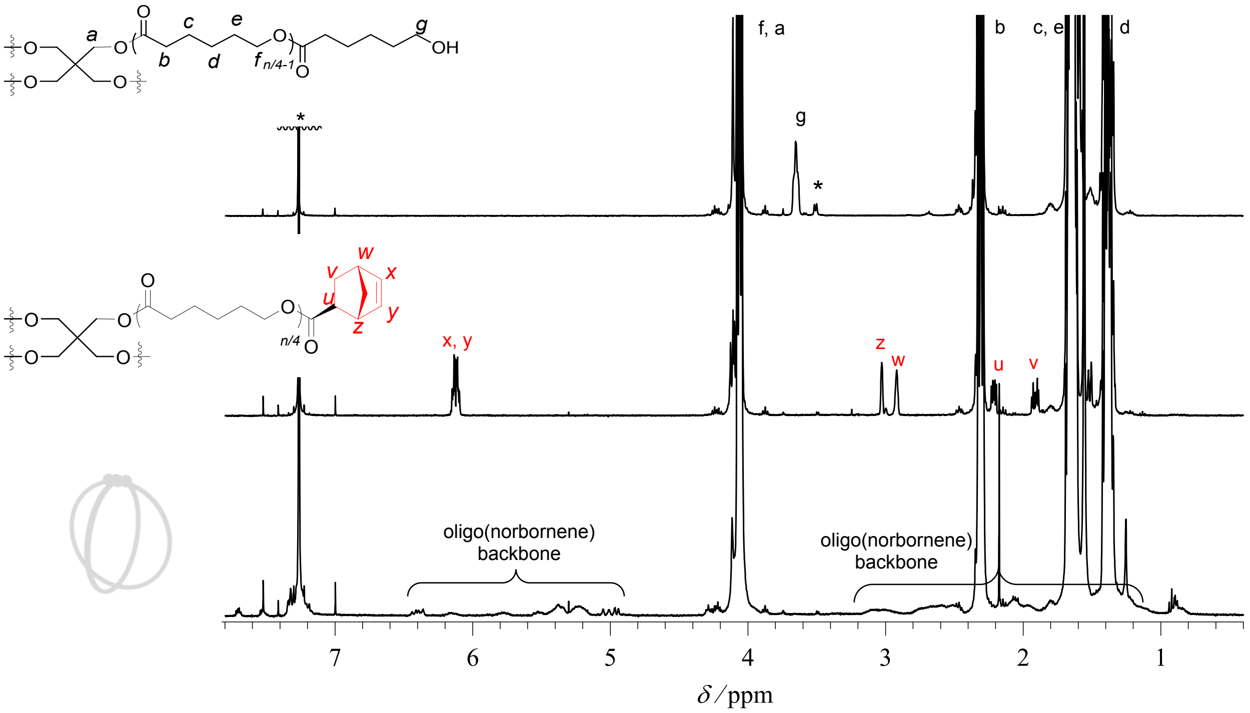

Figure S10. ^{1}H NMR spectra of ***s*-(PCL-OH)$_4$-a** ($M_{\mathrm{n,NMR}}$ = 5,970, $Đ$ = 1.06; upper), **Pre$_4$-a** ($M_{\mathrm{n,NMR}}$ = 5,990, $Đ$ = 1.05; middle), and **cage$_4$-a** ($Đ$ = 1.08; lower) in CDCl$_3$. Asterisks show solvent signals.

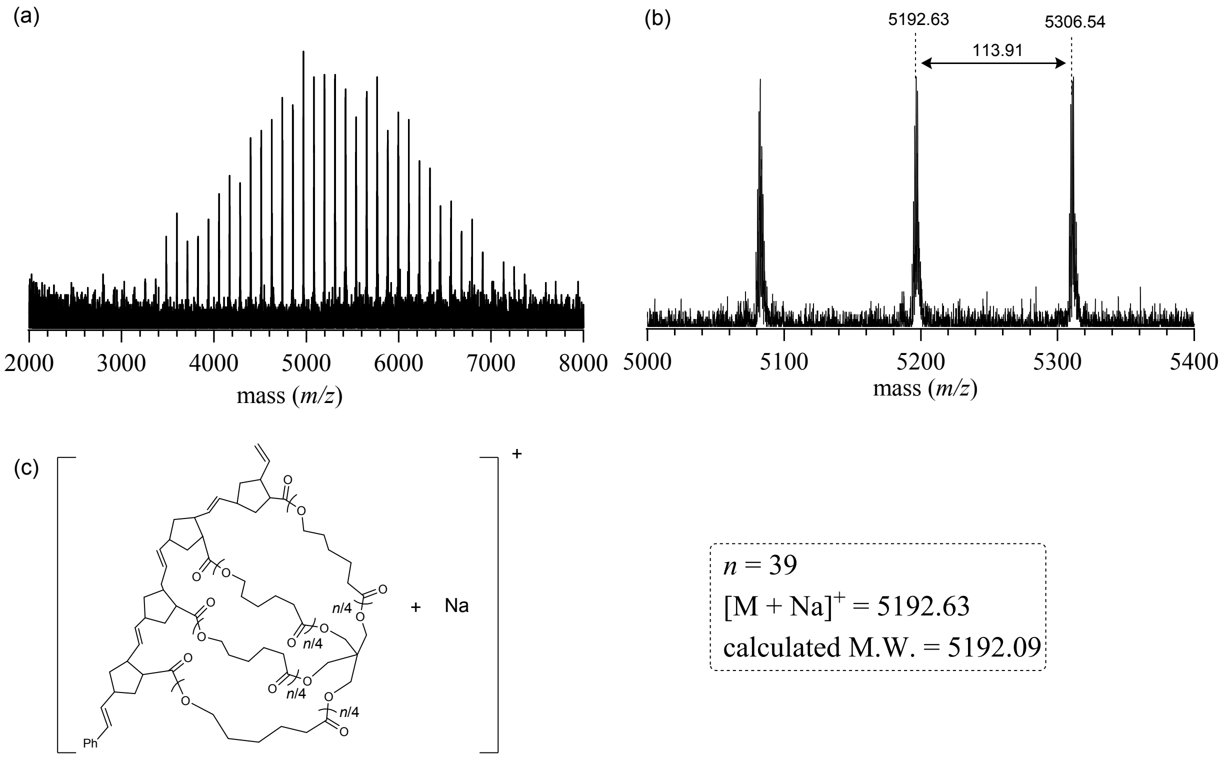

Figure S11. MALDI-TOF MS analysis of the obtained **cage$_4$-a**.

Table S3. Molecular characterization of four-armed macromolecular cages (**cage₄**) and their precursors

Sample	$M_{n,NMR}$ [a]	$M_{n,SEC}$ [b]	$M_{w,MALS}$ [c]	$Đ$ [b]	yield (%)	D_h [c] (nm)	$[\eta]$ [c] (mL g^{-1})	T_m [d] (°C)	X_{WAXD} [e] (%)	L_{ac} [f] (nm)	L_c/L_{ac} [f] (%)
s-(PCL-OH)$_4$-a	5,970	9,290	-	1.06	82.9	-	-	-	-	-	-
Pre$_4$-a	5,990	9,090	6,210	1.05	63.2	5.0	15.4	40.0	31.7	12.3	37.2
cage$_4$-a	-	6,290	7,420	1.08	97.0	4.2	7.4	38.0	32.4	11.1	37.2
s-(PCL-OH)$_4$-b	8,740	12,700	-	1.06	82.3	-	-	-	-	-	-
Pre$_4$-b	8,750	13,500	8,820	1.05	79.5	6.0	19.0	46.0	42.8	12.1	38.3
cage$_4$-b	-	9,590	9,840	1.08	94.0	5.0	10.8	45.2	35.9	11.0	37.9
s-(PCL-OH)$_4$-c	10,400	15,200	-	1.05	84.5	-	-	-	-	-	-
Pre$_4$-c	10,300	15,100	10,100	1.05	39.0	6.6	21.9	48.4	36.2	11.1	40.1
cage$_4$-c	-	10,700	11,800	1.08	91.0	5.6	12.0	47.0	34.4	11.0	38.6

[a] Determined by ^{1}H NMR. [b] Determined by SEC in THF using PSt standards. [c] Determined by SEC-MALS-Visco in THF. $D_h = 2\ R_h = 2(3V_h/4\pi)^{1/3}$; V_h is hydrodynamic volume. V_h was calculated by Einstein–Simha equation ($V_h = M_{w,MALS}[\eta]/2.5N_A$; N_A: Avogadro's number).[d] Determined from a melting peak of the DSC curve. [e] Determined by WAXD at r.t. [f] Determined by SAXS at r.t.

Synthesis of six-armed star-shaped PCL (*s*-(PCL-OH)$_6$)

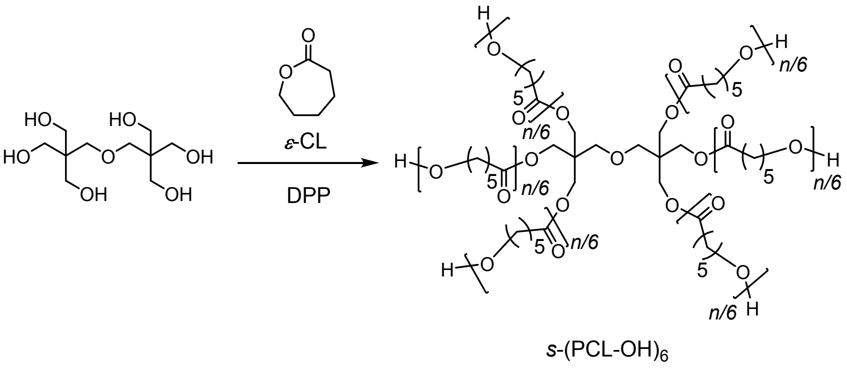

Method A was used for the polymerization of ε-CL (3.00 g, 26.3 mmol), dipentaerythritol (134 mg, 526 μmol) and DPP (6.6 mg, 26 μmol) at 80 °C for 35 min to give ***s*-(PCL-OH)$_6$-a** as a white solid. Yield: 72.0%. ^{1}H NMR (400 MHz, $CDCl_3$): δ (ppm) 4.26-3.91 (m, $-OCO(CH_2)_4CH_2-$, $-OCH_2C(CH_2O\text{-})_3$), 3.70-3.59 (m, $-CH_2OH$), 3.38 (d, J = 11.0, $-OCH_2C(CH_2O-)_3$), 2.51-2.19 ($-OCOCH_2(CH_2)_4-$), 1.86-1.48 ($-OCOCH_2CH_2(CH_2)_3-$, $-OCO(CH_2)_3CH_2CH_2-$), 1.46-1.20 (m, $-OCO(CH_2)_2CH_2(CH_2)_2-$). $M_{n,NMR}$ = 5,300 g mol^{-1} ($CDCl_3$), $M_{n,SEC}$ = 7,000 g mol^{-1} (THF), $Đ$ = 1.09.

Synthesis of *ω*-norbornenyl end-functionalized six-armed star-shaped PCL (Pre$_6$)

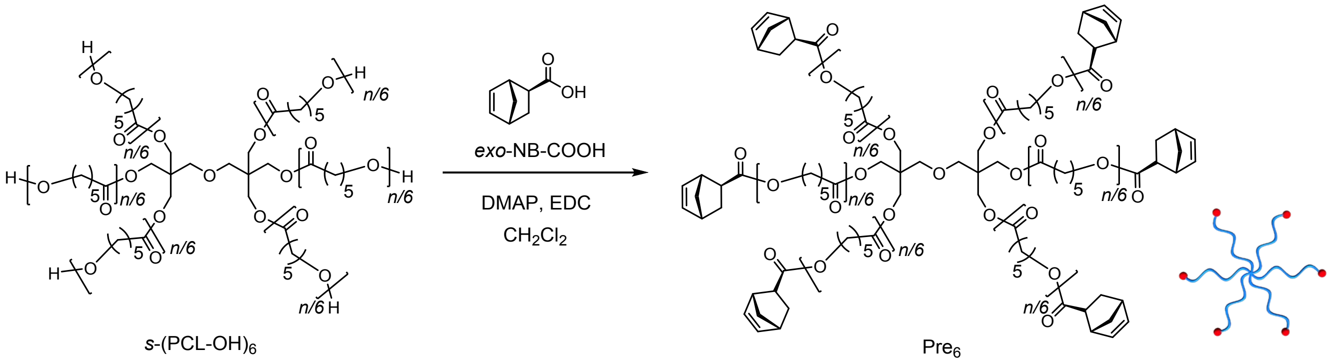

Method B was used for the reaction of ***s*-(PCL-OH)$_4$-a** ($M_{n,NMR}$ = 5,300 g mol^{-1}, 1.00 g, 189 μmol) with *exo*-NB-COOH (313 mg, 2.26 mmol) in the presence of DMAP (415 mg, 3.39 mmol), EDC (650 mg, 3.39 mmol) in CH_2Cl_2 (10 mL) to give **Pre$_6$-a** as a white solid. Yield: 41.0%. ^{1}H NMR (400 MHz, $CDCl_3$): δ (ppm) 6.12 (m, –C*H*=C*H*– in norbornene ring), 4.31-3.85 (m, –OCO(CH_2)$_4$CH_2–, –OCH_2C(CH_2O–)$_3$), 3.09-2.97 (br, –OCH_2C(CH_2O–)$_3$), 3.03 (s, –C*H*–CH–CH_2O– in norbornene ring), 2.92 (s, ––C*H*–CH_2–CH–CH_2O– in norbornene ring), 2.54-2.13 (m, –OCOCH_2(CH_2)$_4$–), 1.96-1.87 (m, –OCO(CH_2)$_2$CH_2(CH_2)$_2$–, bridge head –CH_2– in norbornene ring, *endo*-C*H*– of –CH–CH_2–CH–CH_2O–), 1.77-1.54 (m, –OCOCH$_2$CH_2(CH_2)$_3$–, –OCO(CH_2)$_3$C$H_2$$CH_2$–), 1.48-1.18 (m, –OCO($CH_2$)$_2CH_2$($CH_2$)$_2$–). $M_{n,NMR}$ = 6,030 g mol^{-1} ($CDCl_3$), $M_{n,SEC}$ = 7,760 g mol^{-1} (THF), $Đ$ = 1.06.

Synthesis of six-armed macromolecular cage ($cage_6$) via intramolecular ROMO

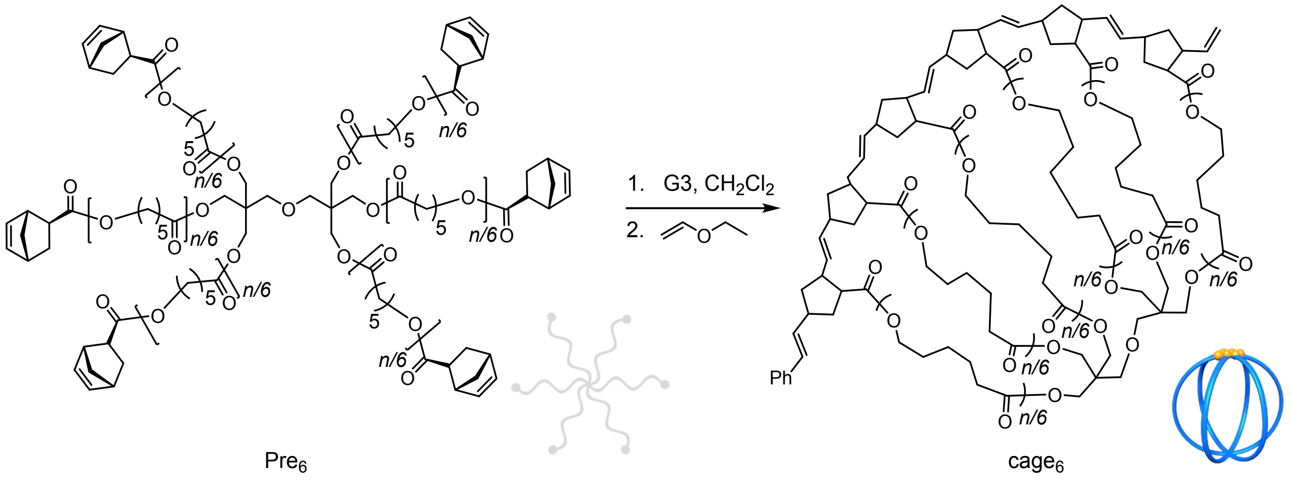

Method C was used for the ROMO of **Pre_6-a** ($M_{n,NMR}$ = 6,030 g mol^{-1}, 30.0 mg, 4.98 μmol, 170 μM in CH_2Cl_2) with G3 (26.4 mg, 29.9 μmol) in CH_2Cl_2 (230 mL) to give **$cage_6$-a** as a pale brown viscous liquid. Yield: 91.0%. ^{1}H NMR (400 MHz, $CDCl_3$): δ (ppm) 7.70, 7.55 (Aromatic), 6.56-4.84 (br, alkenyl of oligo(norbornene) backbone), 4.37-3.72 (m, $-OCO(CH_2)_4CH_2-$, $-OCH_2C(CH_2O-)_3$), 3.58-3.24 (br, $-OCH_2C(CH_2O-)_3$), 3.23-1.85 (br, cyclopentane ring of oligo(norbornene) backbone), 2.40-2.20 (m, $-OCOCH_2(CH_2)_4-$), 1.81-1.50 (m, $-OCOCH_2CH_2(CH_2)_3-$, $-OCO(CH_2)_3CH_2CH_2-$), 1.51-1.14 (m, $-OCO(CH_2)_2CH_2(CH_2)_2-$). $M_{n,SEC}$ = 5,100 g mol^{-1} (THF), $Đ$ = 1.08.

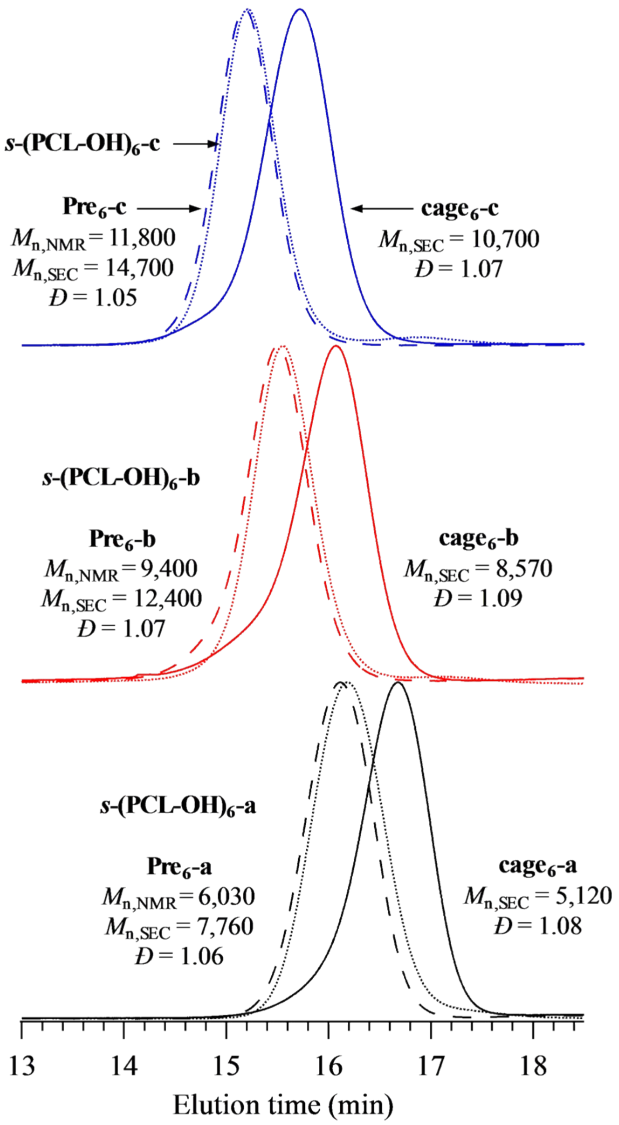

Figure S12. SEC traces of ***s*-(PCL-OH)$_6$**s (dashed line), **Pre$_6$**s (dotted line), and **cage$_6$**s (solid line) with different molecular weight.

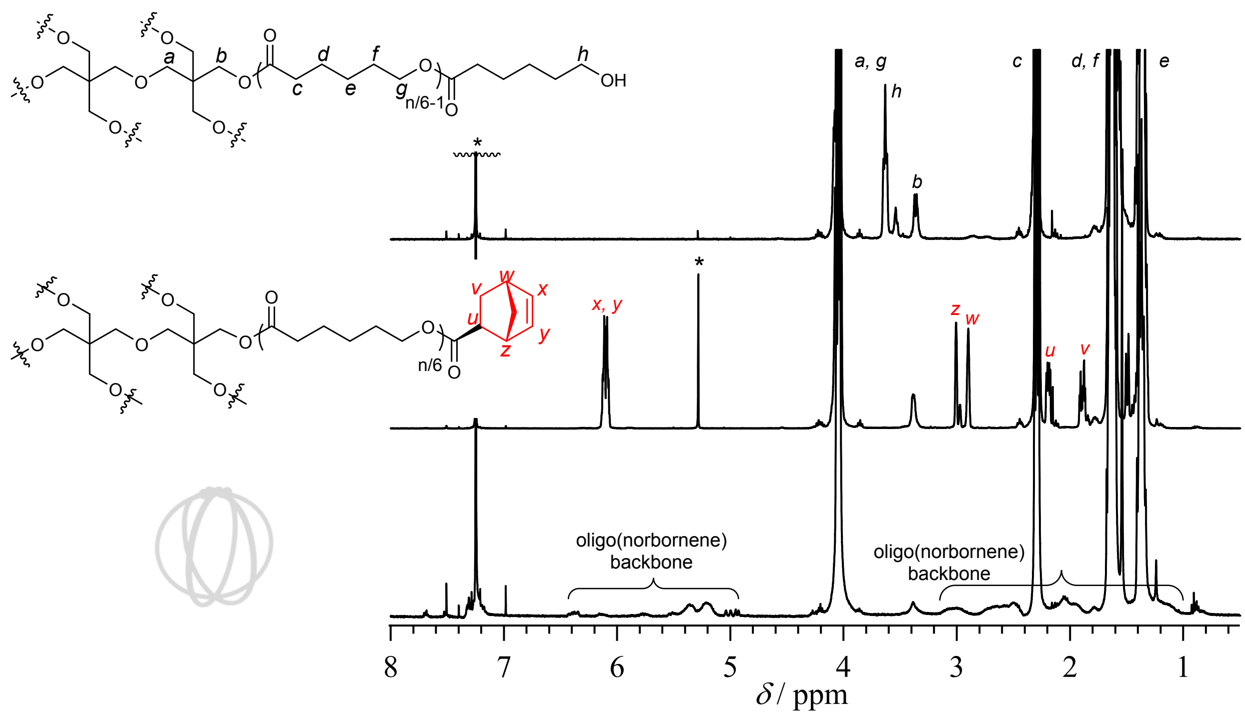

Figure S13. ^{1}H NMR spectra of ***s*-(PCL-OH)$_6$-a** ($M_{n,NMR}$ = 5,300, $Đ$ = 1.09; upper), **Pre$_6$-a** ($M_{n,NMR}$ = 6,030, $Đ$ = 1.06; middle), and **cage$_6$-a** ($Đ$ = 1.08; lower) in $CDCl_3$. Asterisks show solvent signals.

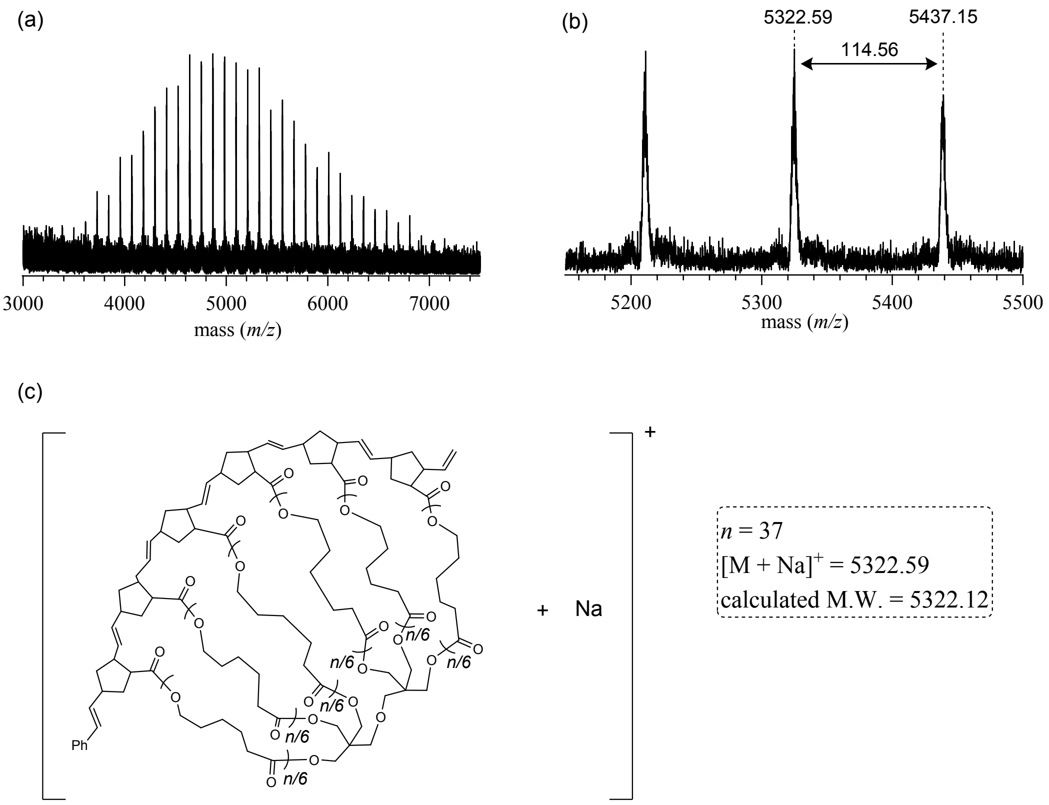

Figure S14. MALDI-TOF MS analysis of the obtained **cage$_6$-a**.

Table S4. Molecular characterization of six-armed macromolecular cages (**cage$_6$**) and their precursors

Sample	$M_{n,NMR}$ [a]	$M_{n,SEC}$ [b]	$M_{w,MALS}$ [c]	$Đ$ [b]	yield (%)	D_h [c] (nm)	$[\eta]$ [c] (mL g^{-1})	T_m [d] (°C)	X_{WAXD} [e] (%)	L_{ac} [f] (nm)	L_c/L_{ac} [f] (%)
s-(PCL-OH)$_6$-a	5,300	7,000	-	1.09	72.0	-	-	-	-	-	-
Pre$_6$-a	6,030	7,760	6,000	1.06	41.1	4.4	11.7	26.4	11.9	N.D.[g]	N.D.[g]
cage$_6$-a	-	5,120	7,950	1.08	91.0	3.8	5.6	N.D.[g]	N.D.[g]	N.D.[g]	N.D.[g]
s-(PCL-OH)$_6$-b	7,760	11,600	-	1.06	67.8	-	-	-	-	-	-
Pre$_6$-b	9,400	12,400	8,880	1.07	49.8	5.6	15.7	39.6	25.7	12.6	38.9
cage$_6$-b	-	8,570	10,900	1.09	98.7	5.0	9.1	31.8	28.2	12.5	37.2
s-(PCL-OH)$_6$-c	10,700	14,400	-	1.04	85.1	-	-	-	-	-	-
Pre$_6$-c	11,800	14,700	11,900	1.05	48.0	6.2	17.5	42.2	33.0	12.6	37.3
cage$_6$-c	-	10,700	12,800	1.07	91.2	5.2	9.8	40.5	29.9	12.5	37.4

[a] Determined by ^{1}H NMR. [b] Determined by SEC in THF using PSt standards. [c] Determined by SEC-MALS-Visco in THF. $D_h = 2\ R_h = 2(3V_h/4\pi)^{1/3}$; V_h is hydrodynamic volume. V_h was calculated by Einstein–Simha equation ($V_h = M_{w,MALS}[\eta]/2.5N_A$; N_A: Avogadro's number).[d] Determined from a melting peak of the DSC curve. [e] Determined by WAXD at r.t. [f] Determined by SAXS at r.t. [g] Not determined.

Synthesis of 5,5'-(((2,2-bis(((2,2,5-trimethyl-1,3-dioxan-5-yl)methoxy)methyl)propane-1,3-diyl)bis(oxy))bis(methylene))bis(2,2,5-trimethyl-1,3-dioxane) (1)

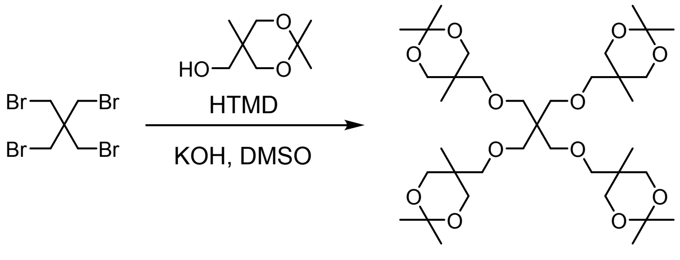

1

HTMD (7.98 g, 49.8 mmol) was added to a stirred solution of KOH (14.0 g, 249 mmol) pentaerythritol tetrabromide (3.87 g, 9.96 mmol) in DMSO (100 mL), and then the solution was stirred at 60 °C for 1 days. After removing the solvent by evaporation, the obtained residue was dissolved in ether and washed with brine. The organic layer was dried over anhydrous Na_2SO_4, filtrated, and then concentrated. The residue was purified by silica gel column chromatography (AcOEt/*n*-hexane = 3/7, R_f = 0.30) to give **1** as a white solid. Yield: 79.3 %. ^{1}H NMR (400 MHz, $CDCl_3$): δ (ppm) 3.81-3.64 (m, 2H, –$CCH_3(CH_2O)_2C$–), 3.60-3.47 (m, 2H, –$CCH_3(CH_2O)_2C$–), 3.45-3.36 (m, 2H, –CH_2OCH_2C–), 3.31 (s, 2H, –CH_2OCH_2C–), 1.40 (d, 6H, J = 12.1, –$C(CH_3)_2$), 0.89 (s, 3H, –CCH_3). ^{13}C NMR (100 MHz, $CDCl_3$): 97.9 (–$C(CH_2)_2$), 74.4, 70.6, 66.8, 34.7, 25.7, 22.1 (–$C(CH_2–)_4$), 18.7 (–CCH_3). HRMS (FD): *m/z* calcd for $C_{37}H_{69}O_{12}$: 705.4789 $[M+H]^+$; found: 705.4776.

Synthesis of 2,2'-(((2,2-bis((3-hydroxy-2-(hydroxymethyl)-2-methylpropoxy)methyl)propane-1,3-diyl)bis(oxy))bis(methylene))bis(2-methylpropane-1,3-diol) (2)

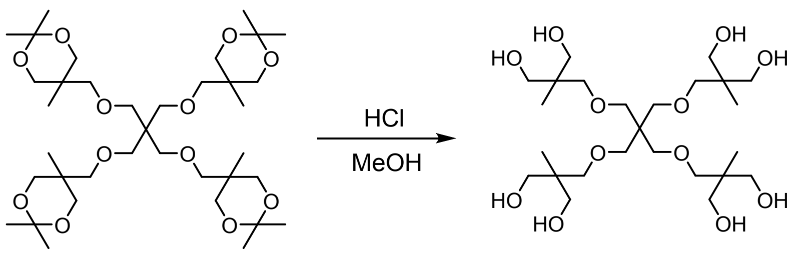

1 **2**

1 (10.2 g, 14.5 mmol) was added to a mixed solvent of MeOH (120 mL) and concentrated hydrochloric acid (5.0 mL), and then the solution was stirred at r.t. for 14 h. After removing the solvent by evaporation, the obtained residue was dried over by a vacuum oven at 150 ºC to give **2** as a white solid. Yield: 90.9 %. ^{1}H NMR (400 MHz, methanol-d_4): δ(ppm) 4.88 (s, 2H, –$CCH_3(CH_2O\mathit{H})_2$), 3.46 (s, 4H, –$CCH_3(C\mathit{H}_2OH)_2$), 3.39 (s, 2H, –$CH_2OC\mathit{H}_2C$–), 3.28 (s, 2H, –$C\mathit{H}_2OCH_2C$–), 0.87 (s, 3H, –$CC\mathit{H}_3(CH_2OH)_2$). ^{13}C NMR (100 MHz, methanol-d_4): 74.2 (–$C(\mathit{C}H_2OH)_2$), 70.5 (–$\mathit{C}CH_3(CH_2OH)_2$), 65.2 (–$CH_2O\mathit{C}H_2C$–), 45.9 (–$\mathit{C}H_2OCH_2C$–), 41.3 (–$\mathit{C}(CH_2–)_4$), 15.9 (–$C\mathit{C}H_3$). HRMS (ESI, in methanol solution): *m/z* calcd for $C_{25}H_{52}O_{12}Na$: 567.3351 $[M+Na]^+$; found: 567.3353.

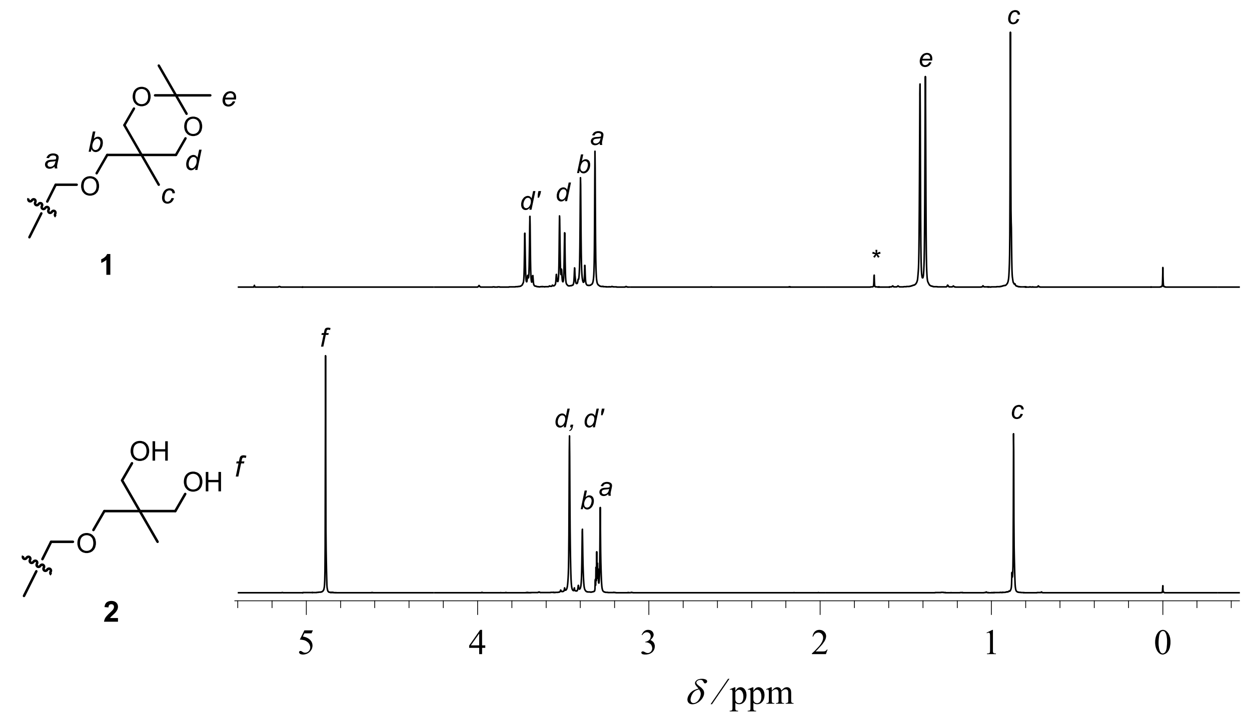

Figure S15. ^{1}H NMR spectra of **1** (upper) and **2** (lower).

Synthesis of eight-armed star-shaped PCL (*s*-(PCL-OH)$_8$)

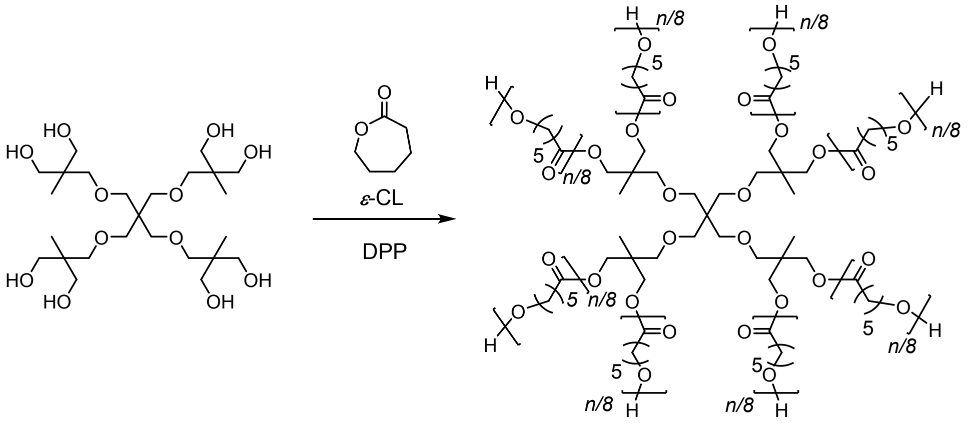

2 *s*-(PCL-OH)$_8$

Method A was used for the polymerization of *ε*-CL (3.00 g, 26.3 mmol) with **2** (286 mg, 525 μmol) and DPP (6.58 mg, 26.3 μmol) at 80 °C for 35 min to give ***s*-(PCL-OH)$_8$-a** as a white solid. Yield: 93.0%. ^{1}H NMR (400 MHz, $CDCl_3$): δ (ppm) 4.15-4.01 (m, $-OCO(CH_2)_4CH_2-$), 3.97 (s, $-CCH_2O-$), 3.65 (q, J = 5.8, $-CH_2OH$), 3.31 (s, $C(CH_2OCH_2-)_4$), 3.22 (s, $C(CH_2OCH_2-)_4$), 2.41-2.24 (m, $-OCOCH_2(CH_2)_4-$), 1.75-1.52 (m, $-OCOCH_2CH_2(CH_2)_3-$, $-OCO(CH_2)_3CH_2CH_2-$), 1.49-1.29 (m, $-OCO(CH_2)_2CH_2(CH_2)_2-$), 0.96, 0.87 (rotamers, $-CCH_3$). $M_{n,NMR}$ = 5,970 g mol^{-1} ($CDCl_3$), $M_{n,SEC}$ = 8,400 g mol^{-1} (THF), $Đ$ = 1.04.

Synthesis of *ω*-norbornenyl end-functionalized eight-armed star-shaped PCL (Pre$_8$)

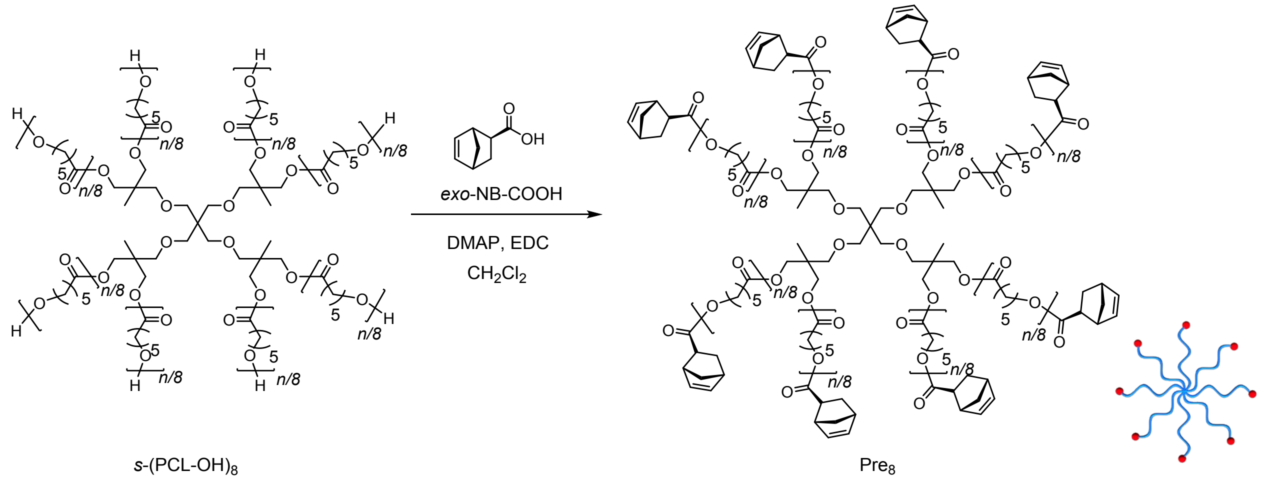

Method B was used for the reaction of ***s*-(PCL-OH)$_8$-a** ($M_{n,NMR}$ = 5,970 g mol^{-1}, 1.00 g, 168 μmol) with *exo*-NB-COOH (370 mg, 2.68 mmol) in the presence of DMAP (491 mg, 4.02 μmol), EDC (770 mg, 4.02 mmol) in CH_2Cl_2 (10 mL) to give **Pre$_8$-a** as a white solid. Yield: 92.1%. ^{1}H NMR (400 MHz, $CDCl_3$): δ (ppm) 6.12 (m, –C*H*=C*H*– in norbornene ring), 4.19-4.01 (m, –OCO(CH_2)$_4$C*H*$_2$–), 3.97 (s, –CC*H*$_2$O–), 3.32 (s, C(C*H*$_2$OCH$_2$–)$_4$), 3.22 (s, C(CH$_2$OC*H*$_2$–)$_4$), 3.03 (s, –C*H*–CH$_2$–CH–CH$_2$O– in norbornene ring), 2.92 (s, –C*H*–CH–CH$_2$O– in norbornene ring), 2.54-2.25 (m, –OCOC*H*$_2$(CH$_2$)$_4$–), 1.97-1.85 (m, H$_3$C*H*$_2$–, –OCO(CH$_2$)$_2$C*H*$_2$(CH$_2$)$_2$–, bridge head –CH$_2$– in norbornene ring, *endo*-C*H*– of –CH–C*H*$_2$–CH–CH$_2$O–), 1.81-1.48 (m, –OCOCH$_2$C*H*$_2$(CH$_2$)$_3$–, –OCO(CH$_2$)$_3$C*H*$_2$CH$_2$–), 1.47-1.21 (m, –OCO(CH$_2$)$_2$C*H*$_2$(CH$_2$)$_2$–), 0.92 (rotamers, –CC*H*$_3$). $M_{n,NMR}$ = 7,300 g mol^{-1} ($CDCl_3$), $M_{n,SEC}$ = 8,930 g mol^{-1} (THF), $Đ$ = 1.04

Synthesis of eight-armed macromolecular cage (cage$_8$) via intramolecular ROMO

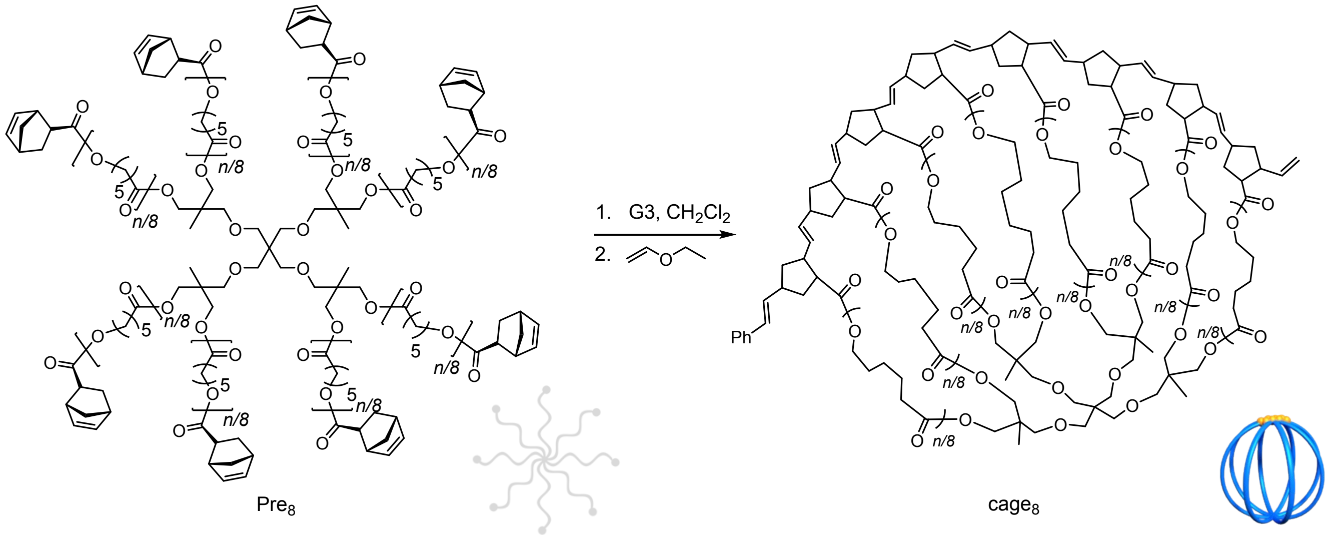

Method C was used for the ROMO of **Pre$_8$-a** ($M_{n,NMR}$ = 7,300 g mol^{-1}, 30.0 mg, 4.11 μmol, 170 μM in CH_2Cl_2) with G3 (21.8 mg, 24.7 μmol) in CH_2Cl_2 (230 mL) to give **cage$_8$-a** as a pale brown viscous liquid. Yield: 98.3%. ^{1}H NMR (400 MHz, $CDCl_3$): δ (ppm) 7.70, 7.53 (Aromatic), 6.57-4.52 (br, alkenyl of poly(norbornene) backbone), 4.16-4.01 (m, $-OCO(CH_2)_4CH_2-$), 3.97 (s, $-CCH_2O-$), 3.40-3.16 (m, $C(CH_2OCH_2-)_4$, $C(CH_2OCH_2-)_4$), 3.15-1.75 (br, cyclopentane ring of poly(norbornene) backbone), 2.41-2.21 (m, $-OCOCH_2(CH_2)_4-$), 1.80-1.51 (m, $-OCOCH_2CH_2(CH_2)_3-$, $-OCO(CH_2)_3CH_2CH_2-$), 1.52-1.05 (m, $-OCO(CH_2)_2CH_2(CH_2)_2-$), 1.04-0.74 (m, $-CCH_3$). $M_{n,SEC}$ = 6,400 g mol^{-1} (THF), $Đ$ = 1.06

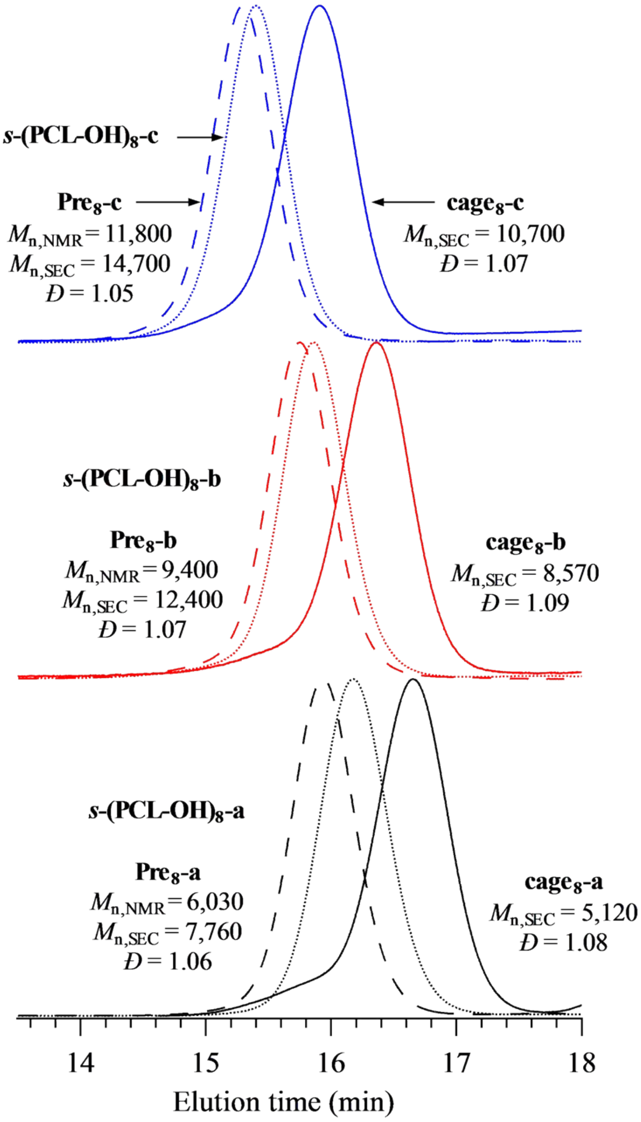

Figure S16. SEC traces of ***s*-(PCL-OH)$_8$**s (dashed line), **Pre$_8$**s (dotted line), and **cage$_8$**s (solid line) with different molecular weight.

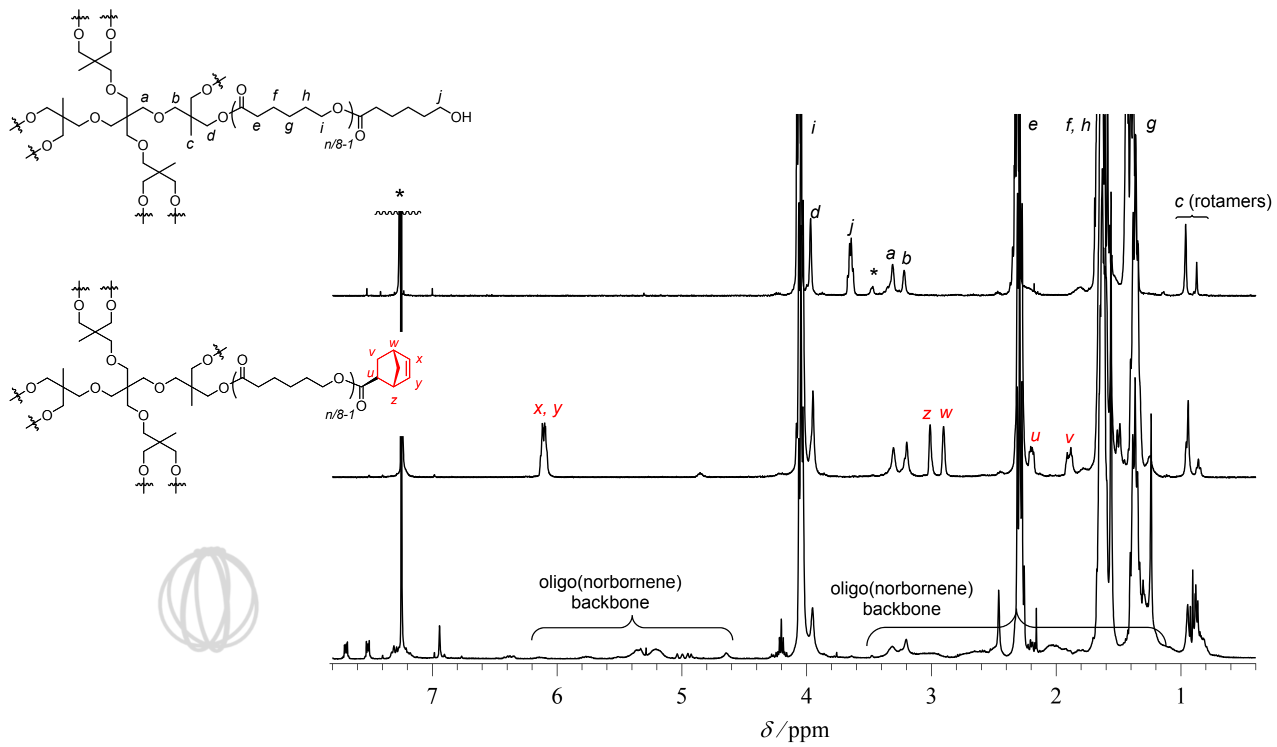

Figure S17. ^{1}H NMR spectra of ***s*-(PCL-OH)$_8$-a** ($M_{n,NMR}$ = 5,970, $Đ$ = 1.04; upper), **Pre$_8$-a** ($M_{n,NMR}$ = 7,300, $Đ$ = 1.04; middle), and **cage$_8$-a** ($Đ$ = 1.06; lower) in $CDCl_3$. Asterisks show solvent signals.

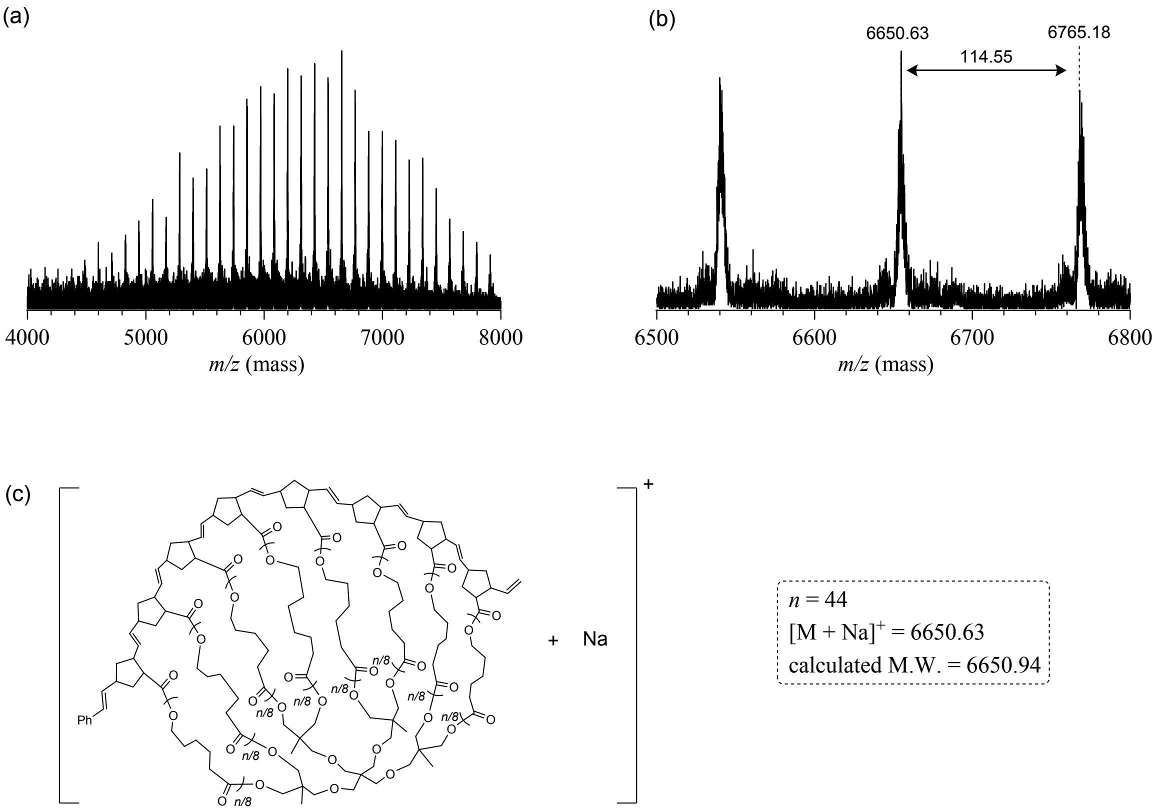

Figure S18. MALDI-TOF MS analysis of the obtained **cage$_8$-a**.

Table S5. Molecular characterization of eight-armed macromolecular cages (**cage$_8$**) and their precursors

Sample	$M_{n,NMR}$ [a]	$M_{n,SEC}$ [b]	$M_{w,MALS}$ [c]	$Đ$ [b]	yield (%)	D_h [c] (nm)	$[\eta]$ [c] (mL g^{-1})	T_m [d] (°C)	X_{WAXD} [e] (%)	L_{ac} [f] (nm)	L_c/L_{ac} [f] (%)
s-(PCL-OH)$_8$-a	5,970	8,400	-	1.04	93.0	-	-	-	-	-	-
Pre$_8$-a	7,300	8,930	7,230	1.04	92.1	4.6	11.1	19.8	N.D.[g]	N.D.[g]	N.D.[g]
cage$_8$-a	-	6,400	8,180	1.06	98.3	3.8	5.4	N.D.	N.D.[g]	N.D.[g]	N.D.[g]
s-(PCL-OH)$_8$-b	8,000	10,500	-	1.04	88.5	-	-		-	-	-
Pre$_8$-b	9,530	11,100	9,280	1.05	72.6	5.4	14.1	38.2	19.5	14.1	38.9
cage$_8$-b	-	8,100	10,300	1.08	97.7	4.6	7.3	N.D.[g]	2.7	N.D.[g]	N.D.[g]
s-(PCL-OH)$_8$-c	11,400	14,600	-	1.04	83.7	-	-		-	-	-
Pre$_8$-c	13,100	15,200	12,800	1.05	64.9	6.6	17.1	42.6	29.2	12.6	37.2
cage$_8$-c	-	11,100	14,300	1.06	84.7	5.6	9.5	31.9	23.3	12.5	37.9

[a] Determined by ^{1}H NMR. [b] Determined by SEC in THF using PSt standards. [c] Determined by SEC-MALS-Visco in THF. $D_h = 2\ R_h = 2(3V_h/4\pi)^{1/3}$; V_h is hydrodynamic volume. V_h was calculated by Einstein–Simha equation ($V_h = M_{w,MALS}[\eta]/2.5N_A$; N_A: Avogadro's number). [d] Determined from a melting peak of the DSC curve. [e] Determined by WAXD at r.t. [f] Determined by SAXS at r.t. [g] Not determined.

S2. Additional Results

S2-1. Systematic evaluation of physical properties for macromolecular cages and their precursors.

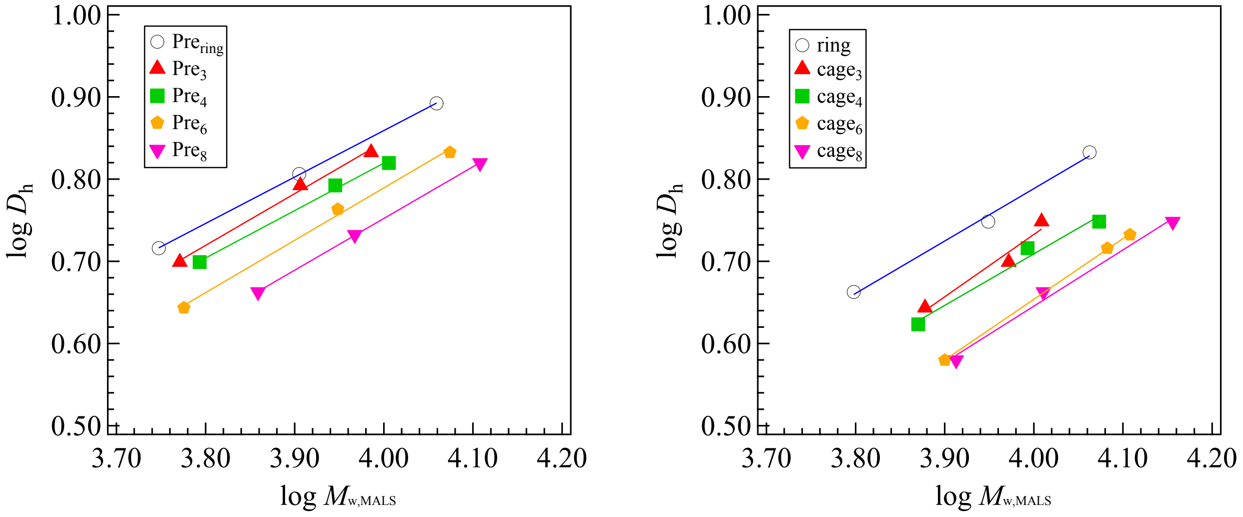

Figure S19. Plots of $M_{w,MALS}$ versus D_h for the precursors (**Pre_{ring}**, **Pre_3**, **Pre_4**, **Pre_6**, and **Pre_8**; left) and the cyclic polymers and macromolecular cages (**ring**, **$cage_3$**, **$cage_4$**, **$cage_6$**, and **$cage_8$**; right).

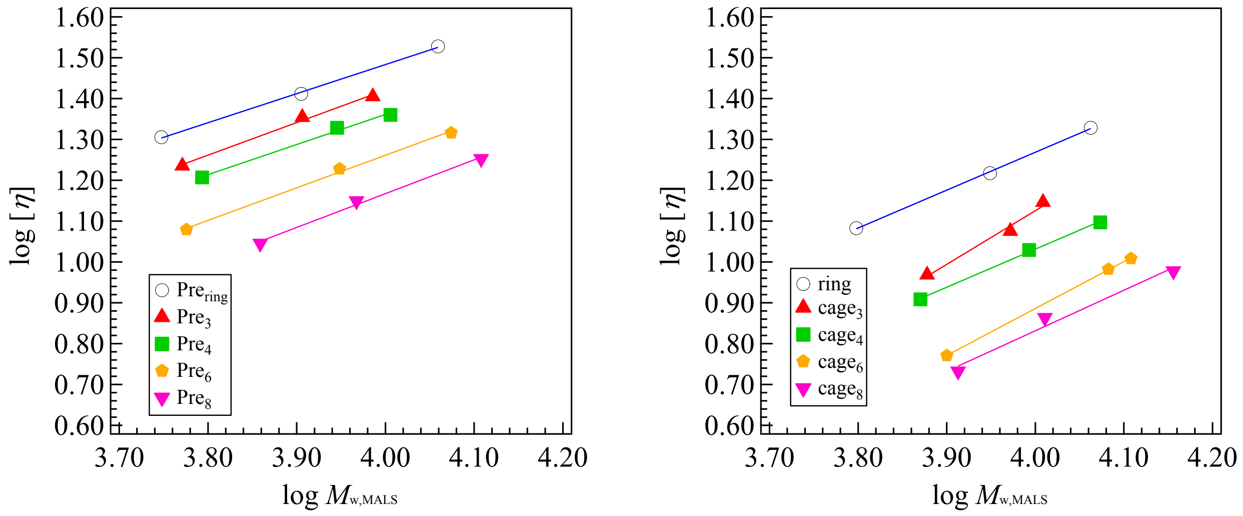

Figure S20. Plots of $M_{w,MALS}$ versus [η] for the precursors (**Pre_{ring}**, **Pre_3**, **Pre_4**, **Pre_6**, and **Pre_8**; left) and the cyclic polymers and macromolecular cages (**ring**, **$cage_3$**, **$cage_4$**, **$cage_6$**, and **$cage_8$**; right).

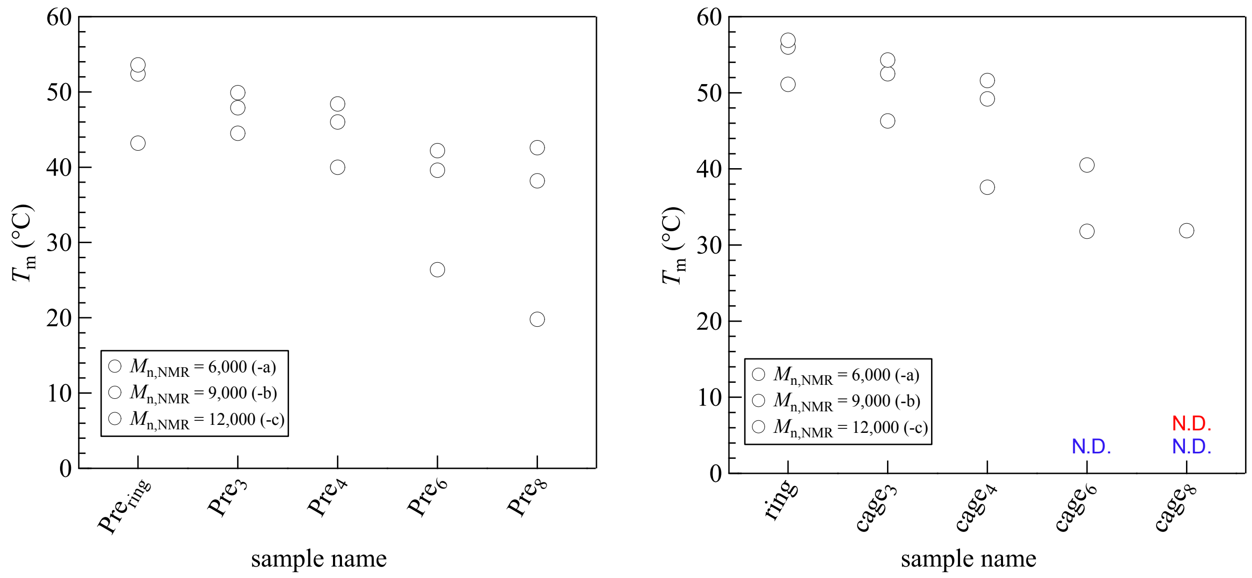

Figure S21. Plots of arm numbers versus melting temperature (T_m) for the precursors (**Pre$_{ring}$**, **Pre$_3$**, **Pre$_4$**, **Pre$_6$**, and **Pre$_8$**; left) and the cyclic polymers and macromolecular cages (**ring**, **cage$_3$**, **cage$_4$**, **cage$_6$**, and **cage$_8$**; right) with varying molecular weight (blue, samples with $M_{n,NMR}$ of ca. 6,000; red, samples with $M_{n,NMR}$ of ca. 9,000; green, samples with $M_{n,NMR}$ of ca. 12,000).

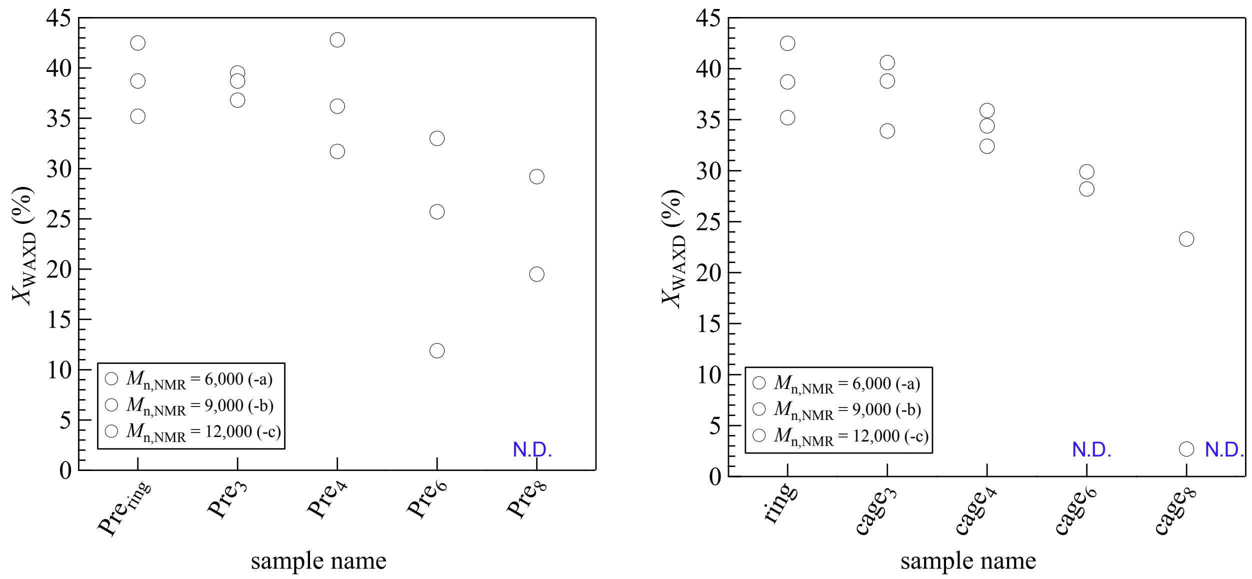

Figure S22. Plots of arm numbers versus crystallinity (X_{WAXD}) for the precursors (**Pre$_{ring}$**, **Pre$_3$**, **Pre$_4$**, **Pre$_6$**, and **Pre$_8$**; left) and the cyclic polymers and macromolecular cages (**ring** and **cage$_3$**, **cage$_4$**, **cage$_6$**, and **cage$_8$**; right) with varying molecular weight (blue: samples with $M_{n,NMR}$ of ca. 6,000; red, samples with $M_{n,NMR}$ of ca. 9,000; green, samples with $M_{n,NMR}$ of ca. 12,000).

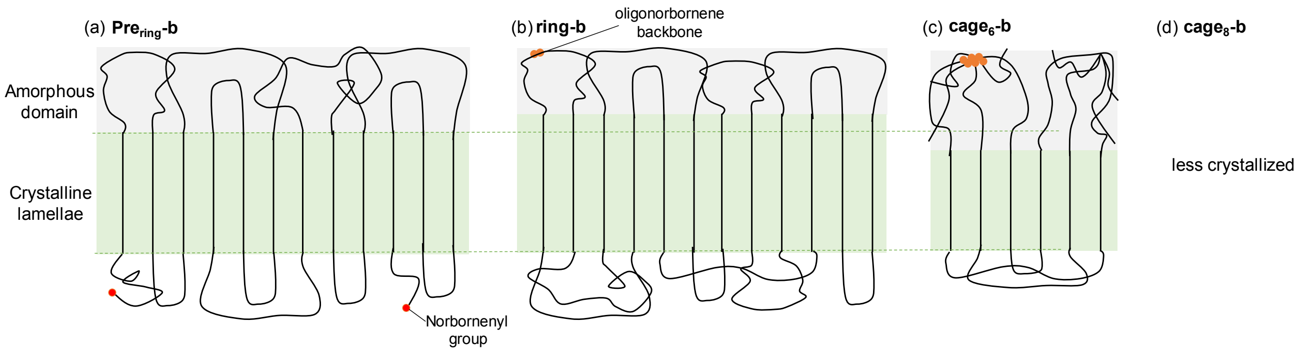

Figure S23. Representative possible crystalline lamellae formation of (a) **Pre$_{ring}$-b**, (b) **ring-b**, (c) **cage$_6$-b**, and (d) **cage$_8$-b** with different lamella thickness, indicated as green domains.

S2-2. SAXS experiment

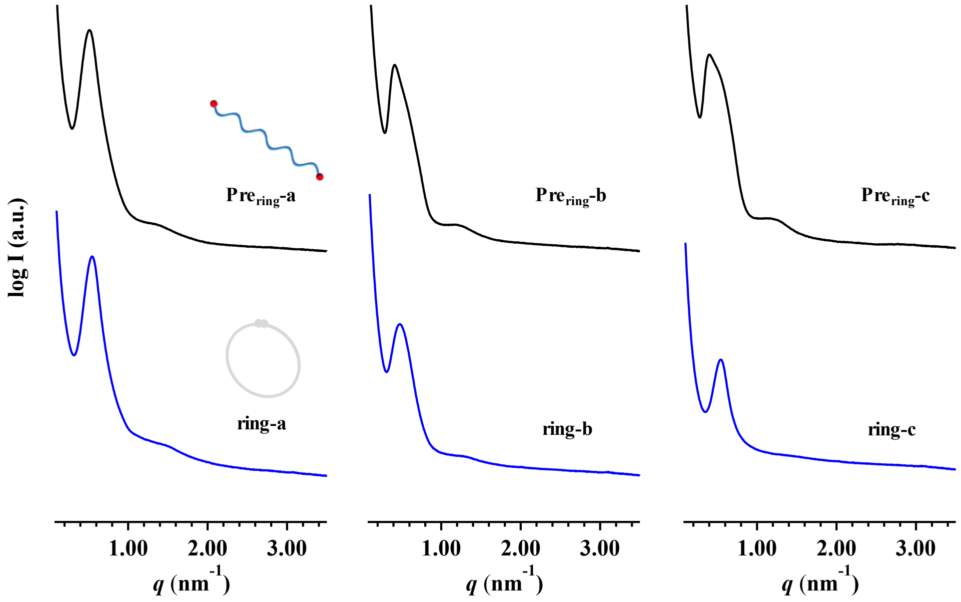

Figure S24. SAXS profiles of the obtained cyclic polymers (**ring**s; blue) and their precursors (**Pre$_{ring}$**s; black) at ambient temperature.

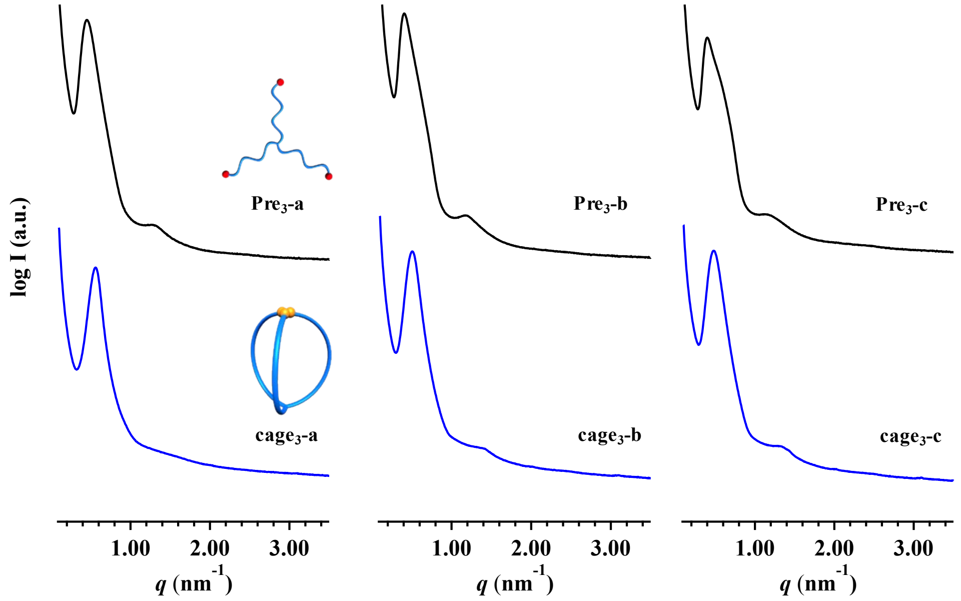

Figure S25. SAXS profiles of the obtained three-armed macromolecular cages (**cage$_3$**s; blue) and their precursors (**Pre$_3$**s; black) at ambient temperature.

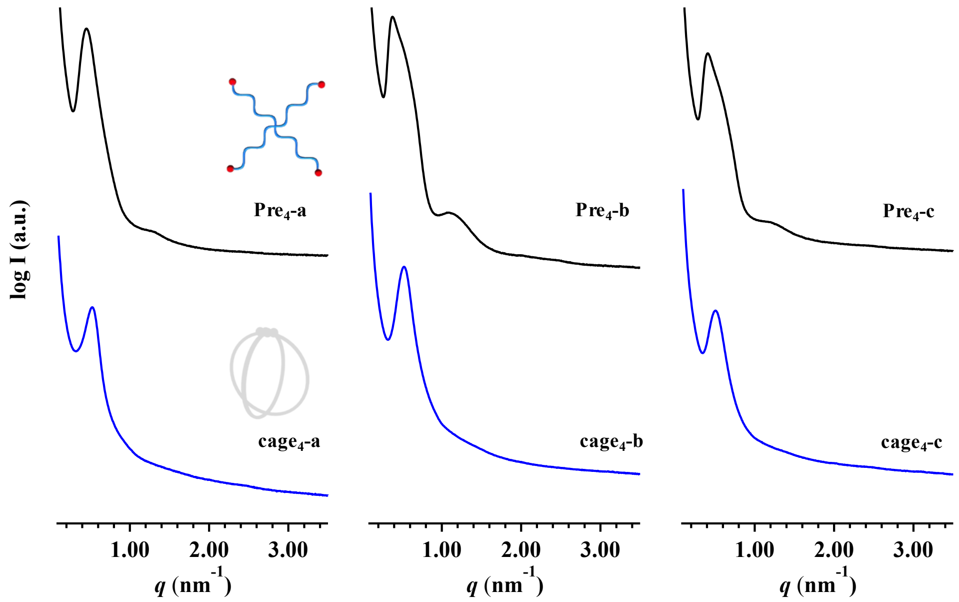

Figure S26. SAXS profiles of the obtained four-armed macromolecular cages (**cage$_4$**s; blue) and their precursors (**Pre$_4$**s; black) at ambient temperature.

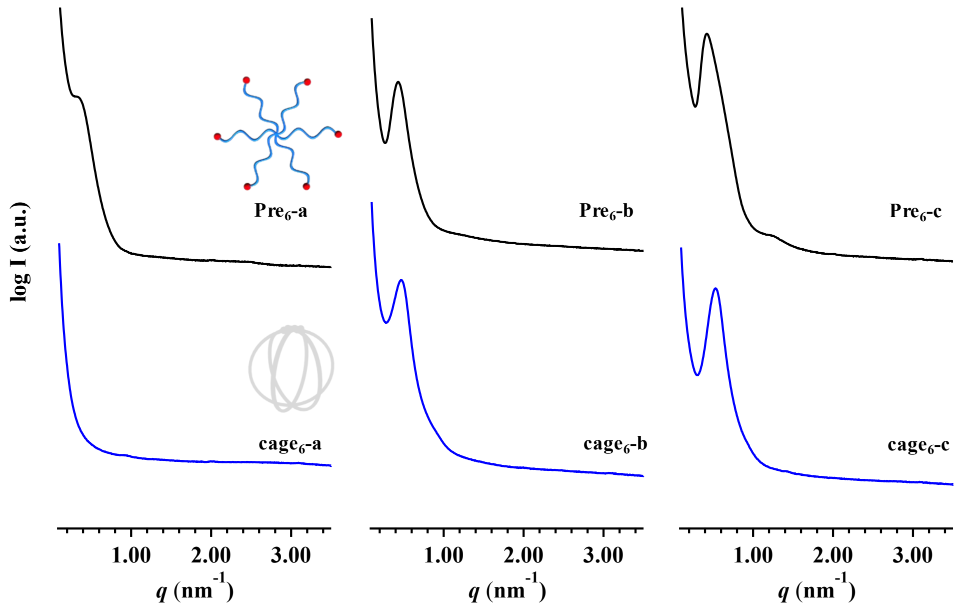

Figure S27. SAXS profiles of the obtained six-armed macromolecular cages (**cage$_6$**s; blue) and their precursors (**Pre$_6$**s; black) at ambient temperature.

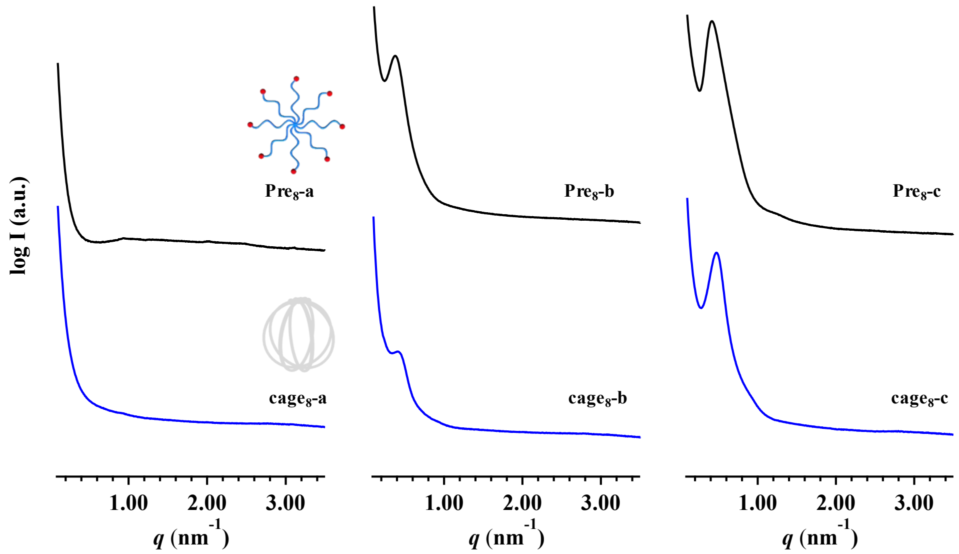

Figure S28. SAXS profiles of the obtained eight-armed macromolecular cages (**cage$_8$**s; blue) and their precursors (**Pre$_8$**s; black) at ambient temperature.

S2-3. WAXD experiment

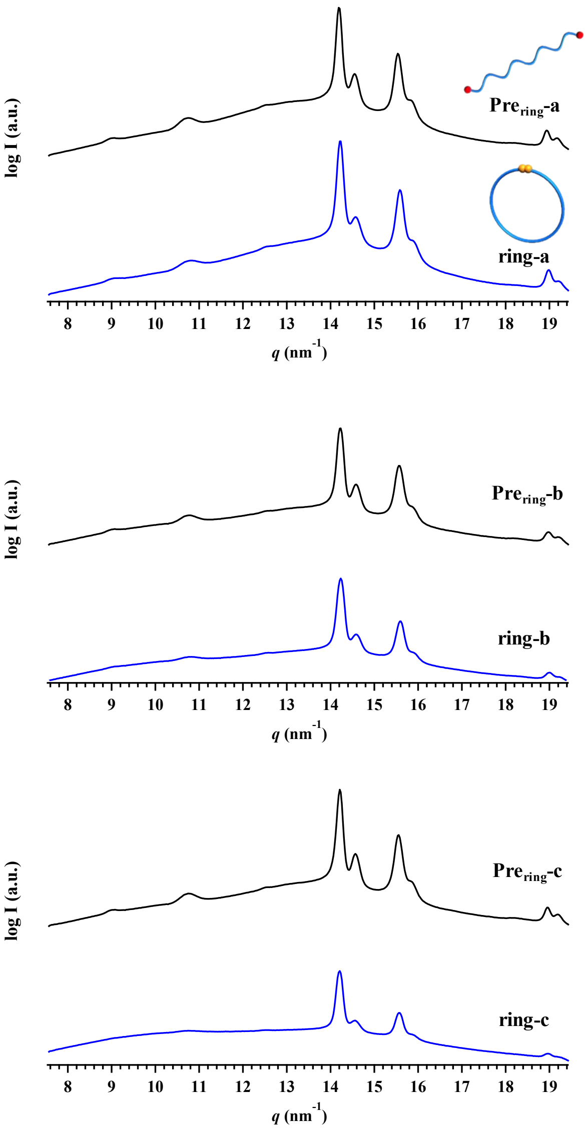

Figure S29. WAXD profiles of the obtained cyclic polymers (**ring**s; blue) and their precursors (**Pre$_{ring}$**s; black) at ambient temperature.

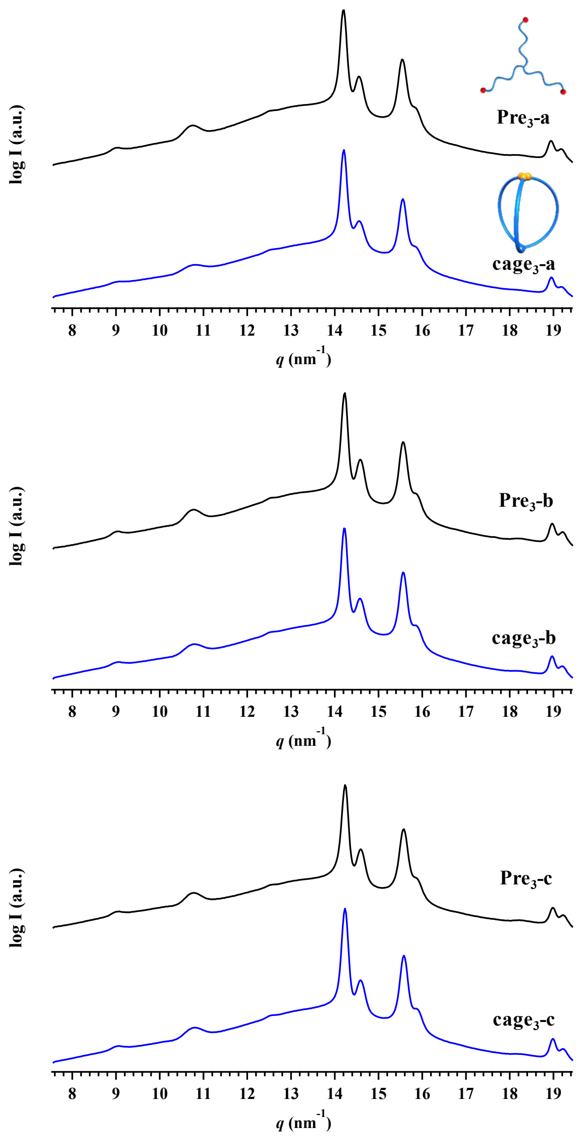

Figure S30. WAXD profiles of the obtained three-armed macromolecular cages (**cage$_3$**s; blue) and their precursors (**Pre$_3$**s; black) at ambient temperature.

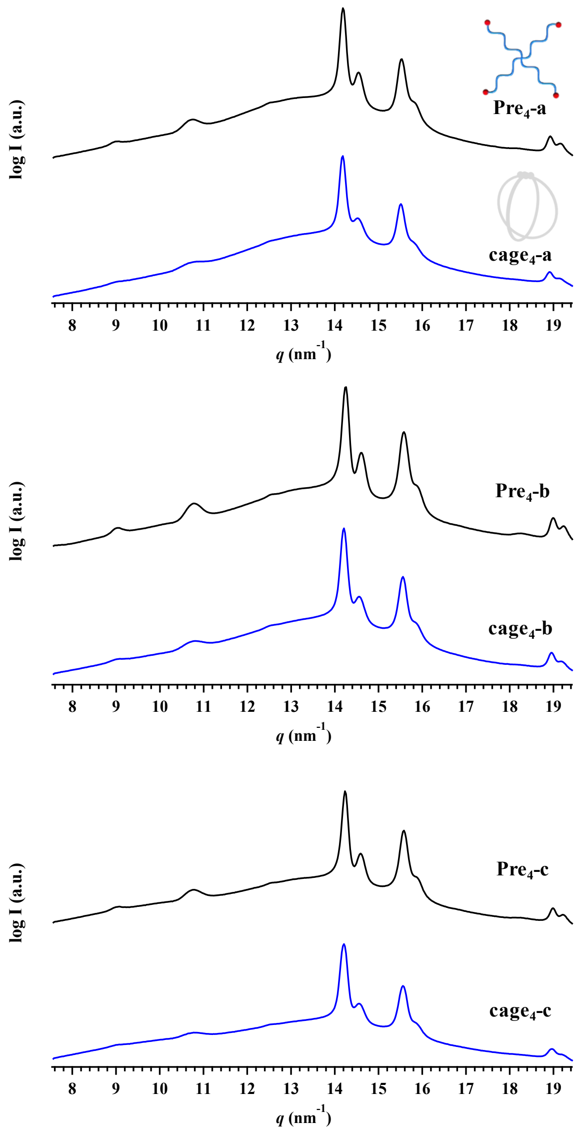

Figure S31. WAXD profiles of the obtained four-armed macromolecular cages (**cage$_4$**s; blue) and their precursors (**Pre$_4$**s; black) at ambient temperature.

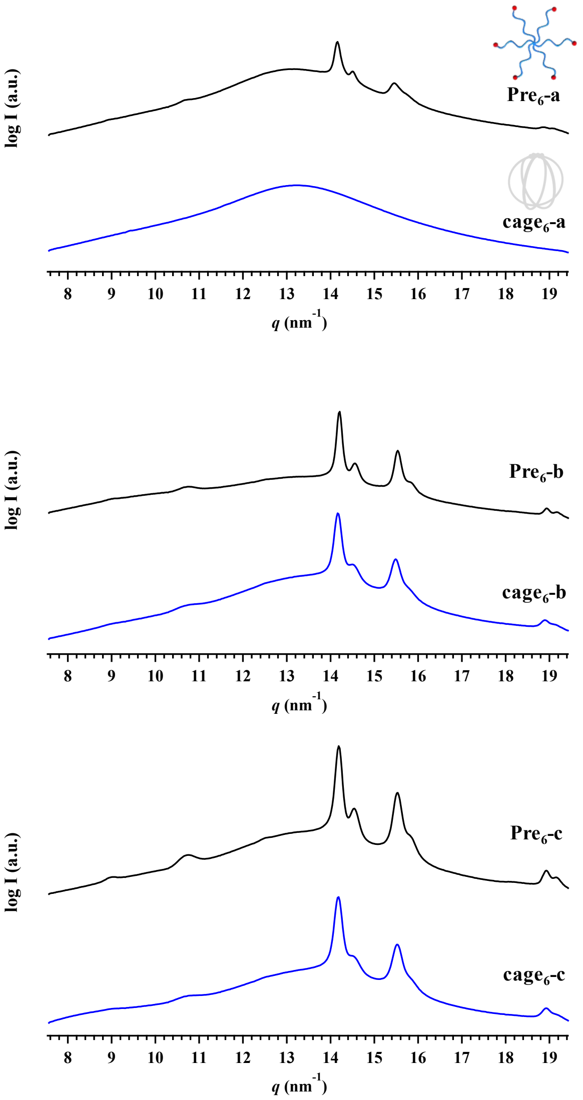

Figure S32. WAXD profiles of the obtained six-armed macromolecular cages (**cage$_6$**s; blue) and their precursors (**Pre$_6$**s; black) at ambient temperature.

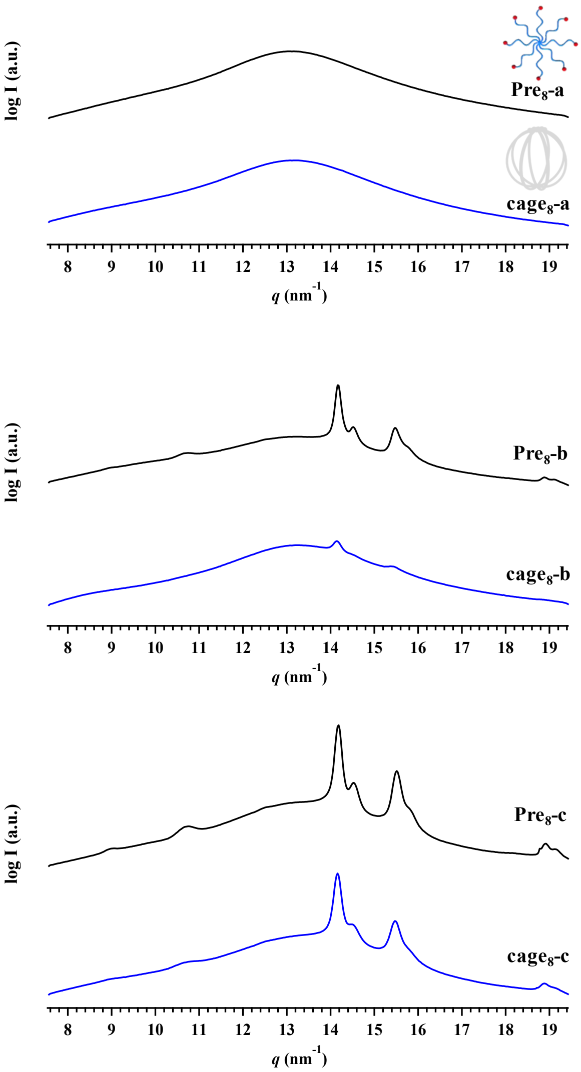

Figure S33. WAXD profiles of the obtained eight-armed macromolecular cages (**cage$_8$**s; blue) and their precursors (**Pre$_8$**s; black) at ambient temperature.

S2-4. Long period estimation by correlation function analysis

The lamellar thickness (L_c) and the long period (L_{ac}) of the PCL crystal were estimated by correlation function analysis of the SAXS profiles of the samples, in accordance with previously reported method[3] using a following equation:

$$\gamma\,(z) = \frac{1}{Q^*}\int_0^{\infty} I(q)q^2cos(qz)dq$$

where Q* is the scattering invariant. The L_c were assigned to the z value of intersection point of linear fitting and horizontal line drew from the first peak as seen in Figure S34, because the crystallinity (X_{WAXD}) of all polymer samples were lower than 50%.

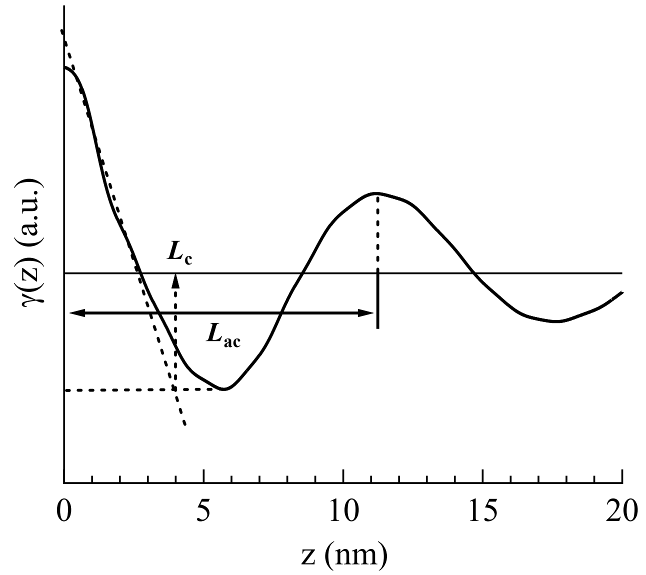

Figure S34. Correlation function analysis of the SAXS profile for **cage$_3$-b**.

S3. References

1. Love, J. A.; Morgan, J. P.; Trnka, T. M.; Grubbs, R. H. A Practical and Highly Active Ruthenium-Based Catalyst that Effects the Cross Metathesis of Acrylonitrile. *Angew. Chem. Int. Ed.* **2002**, *41*, 4035-4037.
2. Ouchi, M.; Inoue, Y.; Wada, K.; Iketani, S. I.; Hakushi, T.; Weber, E. Molecular Design of Crown Ethers. 4. Syntheses and Selective Cation Binding of 16-crown-5 and 19-crown-6 Lariats. *J. Org. Chem.* **1987**, *52*, 2420-2427.
3. Goderis,B.; Reynaers, H.; Koch H. J. M.; Mathot F. B. V. Use of SAXS and Linear Correlation Functions for the Determination of the Crystallinity and Morphology of Semi-crystalline Polymers. Application to Linear Polyethylene. *J. Polym. Sci. Pt B: Polym. Phys.* **1999**, *37*, 1715-1738.
